# A highly contiguous genome assembly reveals sources of genomic novelty in the symbiotic fungus *Rhizophagus irregularis*

**DOI:** 10.1093/g3journal/jkad077

**Published:** 2023-03-31

**Authors:** Bethan F Manley, Jaruwatana S Lotharukpong, Josué Barrera-Redondo, Theo Llewellyn, Gokalp Yildirir, Jana Sperschneider, Nicolas Corradi, Uta Paszkowski, Eric A Miska, Alexandra Dallaire

**Affiliations:** SPUN|Society for the Protection of Underground Networks, 3500 South DuPont Highway, Suite EI-101, Dover, DE 19901, USA; Gurdon Institute, University of Cambridge, Cambridge CB2 1QN, UK; Department of Algal Development and Evolution, Max Planck Institute for Biology, Max-Planck-Ring 5, Tübingen 72076, Germany; Department of Algal Development and Evolution, Max Planck Institute for Biology, Max-Planck-Ring 5, Tübingen 72076, Germany; Comparative Fungal Biology, Royal Botanic Gardens Kew, Jodrell Laboratory, Richmond TW9 3DS, UK; Department of Life Sciences, Imperial College London, London SW7 2AZ, UK; Department of Biology, University of Ottawa, Ottawa, ON, Canada K1N 6N5; Agriculture and Food, Commonwealth Scientific and Industrial Research Organisation, Canberra, ACT 2601, Australia; Department of Biology, University of Ottawa, Ottawa, ON, Canada K1N 6N5; Crop Science Centre, Department of Plant Sciences, University of Cambridge, Cambridge CB3 0LE, UK; Gurdon Institute, University of Cambridge, Cambridge CB2 1QN, UK; Department of Biochemistry, University of Cambridge, Tennis Court Road, Cambridge CB2 1QW, UK; Gurdon Institute, University of Cambridge, Cambridge CB2 1QN, UK; Comparative Fungal Biology, Royal Botanic Gardens Kew, Jodrell Laboratory, Richmond TW9 3DS, UK; Department of Biochemistry, University of Cambridge, Tennis Court Road, Cambridge CB2 1QW, UK

**Keywords:** AMF, arbuscular mycorrhizal fungi, genome assembly, genome evolution, gene birth, chromosome-scale

## Abstract

The root systems of most plant species are aided by the soil-foraging capacities of symbiotic arbuscular mycorrhizal (AM) fungi of the Glomeromycotina subphylum. Despite recent advances in our knowledge of the ecology and molecular biology of this mutualistic symbiosis, our understanding of the AM fungi genome biology is just emerging. Presented here is a close to T2T genome assembly of the model AM fungus *Rhizophagus irregularis DAOM197198*, achieved through Nanopore long-read DNA sequencing and Hi-C data. This haploid genome assembly of *R. irregularis*, alongside short- and long-read RNA-Sequencing data, was used to produce a comprehensive annotation catalog of gene models, repetitive elements, small RNA loci, and DNA cytosine methylome. A phylostratigraphic gene age inference framework revealed that the birth of genes associated with nutrient transporter activity and transmembrane ion transport systems predates the emergence of Glomeromycotina. While nutrient cycling in AM fungi relies on genes that existed in ancestor lineages, a burst of Glomeromycotina-restricted genetic innovation is also detected. Analysis of the chromosomal distribution of genetic and epigenetic features highlights evolutionarily young genomic regions that produce abundant small RNAs, suggesting active RNA-based monitoring of genetic sequences surrounding recently evolved genes. This chromosome-scale view of the genome of an AM fungus genome reveals previously unexplored sources of genomic novelty in an organism evolving under an obligate symbiotic life cycle.

## Introduction

**Fig. 1.**
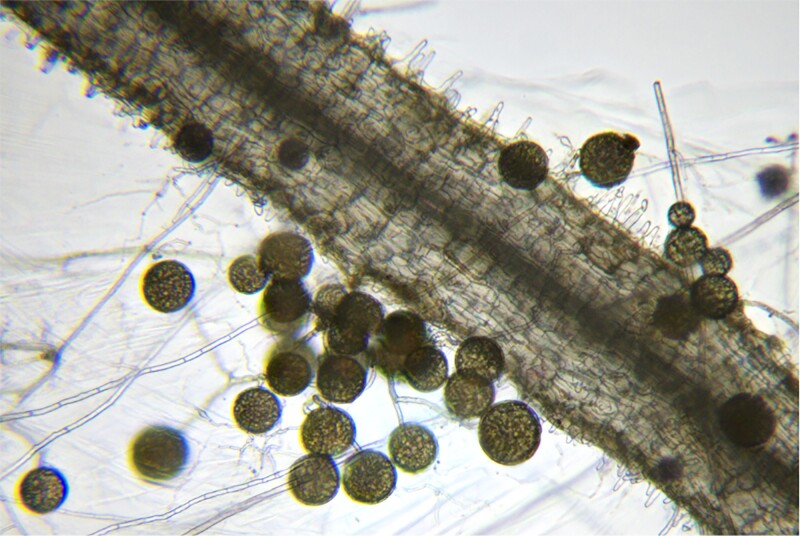
Carrot root with extraradical hyphae and spores of *Rhizophagus irregularis*.

Uprooting almost any terrestrial plant reveals the arbuscular mycorrhizal (AM) symbiosis, a mutually beneficial interaction between most land plant species and members of the fungal Glomeromycotina subphylum (Parniske 2008). AM fungi are multinucleate, obligate symbionts that exist in all terrestrial ecosystems (Davison *et al.* 2015) and engage in symbioses with a wide range of plant species, often simultaneously (Bever 2002). While ecological and molecular mechanistic evidence suggest that the AM symbiosis relies on the reciprocal transfer of organic and inorganic nutrients through a permeable membranous interface (Bonfante and Genre 2010), our understanding of the genomic basis of this symbiotic lifestyle remains limited by the fact that whole-genome sequencing data are available for a limited number of AM species (Trepanier *et al.* 2005; Kobayashi *et al.* 2018; Morin *et al.* 2019; Singh *et al.* 2019, 2021; Sun *et al.* 2019; Venice *et al.* 2020; Malar *et al.* 2021; Montoliu-Nerin *et al.* 2021; Sahraei *et al.* 2022). These include genome assemblies of multiple isolates of the model species, *Rhizophagus irregularis*, and the homokaryotic laboratory strain DAOM197198 (Fig. 1) (Tisserant *et al.* 2013; Lin *et al.* 2014; Chen, Mathieu, *et al.* 2018; Chen, Morin, *et al.* 2018; Maeda *et al.* 2018; Yildirir *et al.* 2022). The most recent genome assembly of DAOM197198 represented a sizeable step-up in genome contiguity and quality (Yildirir *et al.* 2022); however, the contig N50 of 2.3 Mb and quantity of gaps in this assembly is lagging behind recent fungal genome assemblies (Chung *et al.* 2021; Liu *et al*. 2021). The de novo assembly of a reference genome is a crucial step for the genetic research of a given organism. To best support genomic and transcriptomic research, the ideal resource is a fully sequenced, contiguous genomic assembly with few gaps (Church *et al.* 2011; Rhie *et al.* 2021). Recent attention has been paid to the contribution of epigenetic and transposable element landscapes of *R. irregularis* to the adaptation and evolution of this species (Chaturvedi *et al.* 2021; Dallaire *et al.* 2021; Yildirir *et al.* 2022). The production of a higher-quality genome for *R. irregularis* will further enable research into the repetitive landscape and the genomic organization of Glomeromycotina fungi and their relatives, providing crucial insights into the biology and evolutionary history of the AM symbiosis.

This study presents a highly contiguous and near-gapless long-read assembly of the *R. irregularis* isolate DAOM197198 achieved using long Nanopore reads, Hi-C data, and manual curation. Nanopore RNA-Sequencing was generated for *R. irregularis*, producing long reads that span entire transcripts to guide and improve Illumina short-read-based gene model predictions and to enable the annotation of untranslated (UTR) regions, prediction of poly(A) signals, and analysis of poly(A) tail length. Repetitive element, small RNA loci, and DNA cytosine methylome annotations are also provided. These datasets were combined with a tree-of-life scale analysis of gene birth events (Barrera-Redondo *et al.* 2023), which assigns an evolutionary age to protein-coding genes of *R. irregularis* and identifies taxonomically restricted genes that have no detectable homologs in other organisms. This analysis identifies molecular functions that are ancestral to the Glomeromycotina and describes an important gene birth event coinciding with their emergence. The chromosomal distribution of genetic and epigenetic features uncovers evolutionarily young regions of the genome that are potential cradles for new genes and small RNA production.

## Methods

### DNA preparation and sequencing

High-molecular-weight DNA was extracted from 2 g of *R. irregularis* DAOM197198 Grade A spores (Agronutrition) (Schwessinger and McDonald 2017). About 100 mg of ground spore material was resuspended in lysis buffer and processed as indicated. Two successive rounds of cleanup were performed using a 0.45× volume of Ampure XP beads in DNA-Lo-Bind tubes following the manufacturer's protocol. DNA was finally eluted in 50 µL of 10 mM Tris-pH8. DNA quality was assessed by running on a 0.5% agarose gel. Sequencing libraries were prepared using the Oxford Nanopore Rapid DNA sequencing kit SQK-RAD004 and sequenced on MinION flow cells R9.4.1 following the accompanying protocol. Genomic Nanopore reads were basecalled with Guppy Basecalling Software version 5.0.11 + 2b6dbff (Oxford Nanopore Technologies, Limited).

### *Rhizophagus irregularis* DAOM197198 genome assembly and polishing

In total, 9.06 Gb of Nanopore sequence reads was trimmed to remove adapters using Porechop (version 0.2.4), and 1,288,465 of 1,288,893 reads were retained after trimming (99.97%). Following trimming, read N50 was 24,957 bp. The Shasta long-read assembler (shasta-Linux-0.8.0) was then used to produce a raw genome assembly using the parameters --Assembly.consensusCaller Bayesian:guppy-5.0.7-a, --Kmers.k 10, --MinHash.minHashIterationCount 50, --Align.bandExtend 20, --Align.downsamplingFactor 0.1, --ReadGraph.creationMethod 0, –ReadGraph.maxAlignmentCount 12, --ReadGraph.crossStrandMaxDistance 0, --Align.minAlignedFraction 0.3, --Align.minAlignedMarkerCount 60, --Align.maxSkip 50, --Align.maxDrift 30, --Align.maxTrim 30, --MarkerGraph.minCoveragePerStrand 3, --Assembly.iterative, and --Assembly.pruneLength 1500.

The raw assembly was then trimmed of contigs smaller than 500 bp (removing 2 contigs). Subsequent polishing of this trimmed assembly was carried out using the PEPPER-Margin-DeepVariant pipeline as described in Shafin *et al.* (2020, 2021). Broadly, the Nanopore reads described above were aligned against the raw, trimmed *R. irregularis* assembly using minimap2 (parameters: -ax map-ont). About 83.7 Gb of Illumina reads obtained from Maeda *et al.* (2018) was also aligned against this assembly using BWA-MEM with default parameters (Li 2018). Alignments of the Nanopore and Illumina reads produced variant calls that were corrected in the assembly using the PEPPER-Margin-DeepVariant pipeline and Merfin (Formenti *et al.* 2022).

To assemble the telomeric regions of this genome, 1964 reads containing the telomeric repeat TTAGGG_8_ were extracted from trimmed Nanopore reads. These repeat-containing reads were then used to assemble 62 telomeric contigs using Shasta with parameters as described above, with the exception of --Assembly.consensusCaller Bayesian:guppy-5.0.7-a and --Kmers.k 14. The 62 telomeric contigs were polished using the same polishing pipeline as described above, mapping the initial telomere repeat-containing reads and genomic Illumina reads to the telomeric contigs and polishing using the PEPPER-Margin-DeepVariant pipeline. The full genome contigs and the telomeric contigs were then manually fused based on overlapping sequence identified following minimap2 alignment (parameters: -ax map-ont). The QV score of the raw assembly was Q29.49, increasing to Q32.6 following polishing with PEPPER, and finally Q36.27 after polishing with DeepVariant and fusing with separately assembled and polished telomeric contigs.

**Fig. 2.**
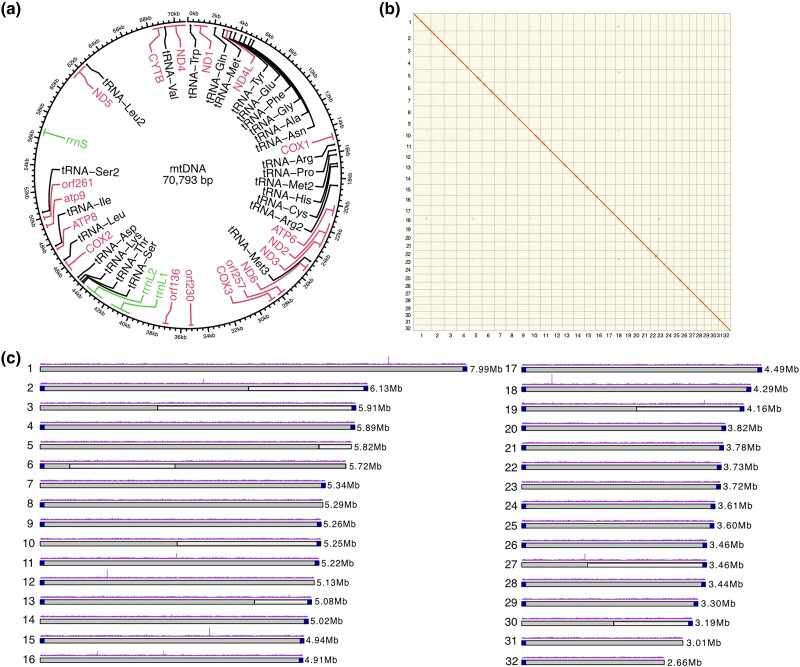
Nuclear and mitochondrial genome assemblies of *Rhizophagus irregularis*. a) Circular map of mitochondrial genome with annotated genes (pink), tRNAs (black), and rRNAs (green). b) Hi-C contact map visualized in PretextView. Chromosomes are displayed in size order from left to right (1–32). c) Physical map of 32 chromosomes numbered according to size (Mb). Grey coloring of the ideogram highlights contigs that were scaffolded together. Telomeric sequences are represented by dark blue squares at the ends of ideograms. Nanopore read coverage is shown as a purple histogram.

The assembly process resulted in the assembly of a complete, circular mitochondrial genome of 70,793 bp. The circularity of the mitochondrial assembly graph was visualized using Bandage (Supplementary Fig. 1a) (Wick *et al.* 2015). MitoHifi v.2.2 (Laslett and Canback 2008; Allio *et al.* 2020; Uliano-Silva *et al.* 2021) was used to annotate the mitochondrial genome (Fig. 2a). This mitochondrial genome was removed from the nuclear genome assembly for manual curation. Hi-C read data for *R. irregularis* DAOM197198 (Yildirir *et al.* 2022) were aligned to the remaining 42 contigs using BWA-mem (Li and Durbin 2009) and the subsequent alignment file was used to produce a PretextView Map (Harry 2020). The PretextView Hi-C contact map and the assembled contigs were manually curated (as described in Howe *et al.* 2021) to produce chromosome-scale scaffolds (Table 1).

**Table 1.** Summary of *Rhizophagus irregularis* genome assemblies.

Accession	GCA_026210795.1	GCA_020716725.1	GCA_002897155.2	GCA_000439145.3
Reference	This study	Yildirir *et al*. (2022)	Maeda *et al*. (2018)	Chen, Morin, *et al*. (2018)
Number of contigs	42	107	210	5,983
Contig N50 (bp)	3,900,757	2,312,895	2,308,129	49,632
Number of scaffolds	32	33	—	1,111
Scaffold N50 (bp)	5,085,394	4,960,142	—	336,373
Number of gaps	10	74	—	7,601
Assembly size (bp)	146,773,001	147,209,168	149,746,764	136,726,313
Number of genes	30,209	26,634	41,572	26,183

### Quality assessment of the *R. irregularis* DAOM 197198 assembly

The genome assembly was scored by BUSCO version 5.2.2 (Simao *et al.* 2015) as 95.8% complete using the fungi_odb10 database. In total, 726 complete BUSCOs were identified out of a total of 758 BUSCO groups searched, of which 13 were duplicated. All trimmed Nanopore reads were mapped to the assembly using Minimap2 (parameters: -ax -map-ont) (Li 2018), resulting in the mapping of 1,279,771 reads to the final assembly (99.32% of total trimmed reads). Mosdepth was used to examine the cumulative distribution of read coverage for each contig. Average Nanopore read coverage was highly uniform, between 77 and 85× across all nuclear contigs (Supplementary Fig. 1b). To assess Illumina read coverage uniformity, BWA-MEM was used to align Illumina genomic DNA reads (Maeda *et al.* 2018) to the assembly, with 200,768,646 of 211,520,841 (94.61%) reads mapping successfully. Average Illumina read coverage of contigs identified through mosdepth (https://github.com/brentp/mosdepth) was again uniform, all contigs displayed coverage between 227× and 232× (Supplementary Fig. 1b). Additionally, a BLASTn analysis (parameters: -task megablast, -max_target_seqs 25, -culling_limit 2, -evalue 1e-25) was carried out on the assembly and the best hit for each contig was *R. irregularis.* A whole-genome pairwise alignment between the current assembly and the Yildirir *et al.* (2022) assembly was generated using the nucmer (version 4.0.0) script from mummer3 (Marcais *et al.* 2018), followed by visualization of the output delta files using Dot (https://dot.sandbox.bio) (Supplementary Fig. 1c).

### Genome annotation

*Rhizophagus irregularis* DAOM197198 RNA samples (plates of 50,000 spores/sample) used for genome annotation and the protocol for the production of rice exudates to treat spore plates were the same as described previously (Dallaire *et al.* 2021). The Illumina RNA-Seq samples used were an untreated spore plate, a 24-hour rice exudate-treated sample, a 48-hour rice exudate-treated sample (48e_1), and a sample of *R. irregularis-*colonized maize root (growth conditions with RNA extraction as described for rice plants in Dallaire *et al.* 2021). Additional Illumina RNA-Seq samples from a further experiment described in the same publication used for genome annotation were 2 *Nicotiana benthamiana* root samples colonized by *R. irregularis* and 2 germinated spore samples. Short-read library preparation, sequencing, and adapter trimming were carried out on paired-end polyA+ RNA by Novogene UK Co. Ltd. with read lengths of 150 bp.

The TrimGalore!-0.6.6 wrapper script for Cutadapt (Martin 2011) was used for quality and adapter trimming of all short-read fastq files (parameters: --length 36 -q 20 --stringency 1 -e 0.1 --paired --phred33). For alignment of the Illumina RNA-Seq files to the soft-masked genome assembly, STAR (version 2.7.6a) was used (parameters: --outFilterMultimapNmax 20) (alignment statistics in Table 2) (Dobin *et al.* 2013). Output BAM files from this STAR alignment were used as input for BRAKER 2.1.5 (parameters: –gff3 –fungus –softmasking) (Bruna *et al.* 2021). Protein domains were predicted from BRAKER2 models using InterProScan 5.55–88.0 (Jones *et al.* 2014) and were manually curated to remove genes with transposon-related protein domains, leading to the Illumina-based gene annotation presented in this study.

**Table 2.** RNA-Seq datasets used for gene annotation.

Seq. technology	Library name	Reads mapped	Data access	Reference
Illumina PE150	0h_untreated_rep1 (spores)	22,648,424 (93.92%)	GSE172187	Dallaire *et al*. (2021)
	24h_exudate_rep1 (spores)	25,766,642 (94.62%)		
	48h_exudate_rep1 (spores)	22,237,703 (93.65%)		
	Colonized maize	1,093,801 (4.88%)		This study
Illumina PE75	Spores2	28,053,926 (85.42%)	PRJNA722386	Dallaire *et al*. (2021)
	Spores3	45,435,299 (76.34%)		
	Myc2 (*N. benthamiana*)	28,251,335 (11.97%)		
	Myc3 (*N. benthamiana*)	23,216,161 (9.66%)		
Nanopore	Spores (0 h untreated)	5,003,748 (91.61%)	*TBA*	This study
	Spores (48 h exudate)			

To produce the long-read RNA-Seq data used to refine Illumina-based gene models, Nanopore RNA-Seq was carried out using a sample of *R. irregularis* DAOM197198 pre-germinated spore plate (50,000 spores/plate) (Table 2 and described below). About 1 µg of total RNA from 3 samples was individually poly(A) selected using the NEBNext Poly(A) mRNA Magnetic Isolation Module (NEB #E7490). Poly(A)+ concentration and rRNA depletion were assessed using Qubit and TapeStation. Approximately 20 ng from each sample (total of 70 ng of poly(A)-selected RNA) was barcoded using the Nanopore PCR-cDNA barcoding kit (Kit SQK-PCB109). PCR was performed with 14 cycles and 6.5-minute extensions. About 33.3 fmol of each barcoded sample was pooled together and prepared for sequencing on an R9.4.1 flow cell. Sequence reads were demultiplexed and basecalled using Guppy Basecalling Software version 5.0.11 + 2b6dbff (Oxford Nanopore Technologies, Limited). Following basecalling, Pychopper version 2.5 was used to trim the reads and rescue fused reads. These reads were provided as evidence to the update script of funannotate version 1.8.7 (https://zenodo.org/record/4054262#.Yv4hJy8w3AY), which uses PASA (Haas *et al.* 2003, 2008), to refine gene models of the Illumina-based gene annotation; to predict 5′UTR, 3′UTR, and polyadenylation signal sequences; and to extract poly(A) tail sequences. Transposon-related protein domains were removed from the updated gene models, leading to the final Illumina + Nanopore-based gene annotation presented in this study.

Illumina-based and Illumina + Nanopore-based gene models were functionally annotated separately using funannotate version 1.8.7, ran with the BUSCO database “fungi” and the UniProt DB version 2022_01, and with protein domain prediction evidence from (1) InterProScan 5.55–88.0 (Jones *et al.* 2014), (2) eggnog-mapper v2.1.7 (with more-sensitive mode, corresponding to diamond.2.0.8's very-sensitive mode (Huerta-Cepas *et al.* 2019; Buchfink *et al.* 2021; Cantalapiedra *et al.* 2021)), and (3) Secondary metabolism and transmembrane domain prediction using antiSMASH version 6.0.1 (Blin *et al.* 2021). Gene annotations were scored by BUSCO with the fungi_odb9 database. GO terms associated with the Illumina + Nanopore-based annotation were processed with g:Profiler's GMT tool, and GO term analyses were performed using the g:Profiler web server (Raudvere *et al.* 2019) using the token “gp__xfGY_dQeI_yx4,” or the GMT file provided as Supplemental File.

### Repeat and transposable element annotation

Repeats were modeled using EDTA (parameter --sensitive 1) (Ou *et al.* 2019). *Rhizophagus irregularis* multi-copy coding genes sometimes get detected as repetitive and wrongly end up in repeat libraries. Protein domains were, therefore, predicted from repeat consensus sequences using InterProScan 5.55–88.0, and consensus sequences containing gene-related InterPro domains were filtered out. The remaining consensus sequences were used to mask the genome assembly using RepeatMasker (parameters -s -no_is -norna -nolow -div 40) (Smit *et al.* 2015).

### Small RNA annotation and quantification

In total, 70,956,710 small RNA-Seq reads from 2 replicates of oxidized and 2 replicates of column-purified spore RNA (Dallaire *et al.* 2021) were used to run ShortStack (Axtell 2013) (parameters --dicermin 20 --dicermax 27 --foldsize 300 --pad 200 --mincov 10.0 rpm --strand_cutoff 0.8 --mmap r).

### DNA methylation basecalling

About 161 Gb of raw FAST5 files obtained from 3 R9.4.1 flow cells was basecalled with Guppy Basecalling Software version 5.0.11 + 2b6dbff, producing 985,449 reads which were successfully processed by tombo (Stoiber *et al.* 2017) and used by DeepSignal2 (Ni *et al.* 2019) to extract CG motifs and to call modifications using a human model (model.dp2.CG.R9.4_1D.human_hx1.bn17_sn16.both_bilstm.b17_s16_epoch4.ckpt).

### Phylostratigraphy analyses

GenEra (Barrera-Redondo *et al.* 2023) was run using DIAMOND in ultra-sensitive mode (Buchfink *et al.* 2021). An *E*-value threshold of 1E^−5^ was chosen to balance the detection of distant homologs while minimizing the amount of false positives against the NR database (Barrera-Redondo *et al.* 2023). Taxonomy IDs used for the focal species are 50,956 for *Geosiphon pyriformis*, 4,874 for *Gigaspora margarita*, 1,432,141 for *R. irregularis*, 101,101 for *Dissophora decumbens*, 1,314,771 for *Mortierella elongata*, 64,574 for *Radiomyces spectabilis*, and 4,837 for *Phycomyces blakesleeanus*. Genes with taxonomic representativeness scores below 30% were flagged as possible contamination or horizontal gene transfer and were not included in subsequent analyses. Some phyloranks were corrected: strain level ranks were moved to species level (“*R. irregularis* DAOM 197198” to “*R. irregularis*” and “*Linnemannia elongata AG-77*” to “*Linnemannia elongata*” and “Fungi *incertae sedis*” was moved to the kingdom level “Fungi”). Several phyloranks were collapsed due to insufficient genomic data (Supplementary Fig. 2a) or unresolved phylogenetic placement of subphyla (Supplementary Fig. 2, b–d). GenEra's homology detection failure test (Weisman *et al.* 2020; Barrera-Redondo *et al.* 2023) was run by using the pairwise evolutionary distances from a phylogenomic tree (Li *et al.* 2021) to obtain a list of genes in *R. irregularis* whose ages cannot be explained by gene untraceability from the genus to the kingdom phyloranks.

### Chromosomal distribution of genomic features and expression

Nanopore RNA-Seq reads were trimmed of adapters and cleaned with seqclean (Chen *et al.* 2007) to remove a percentage of undetermined bases, polyA tails, overall low complexity sequences, and short terminal matches. Cleaned sequences were then mapped using minimap2 (options -G max intron length = 3000, -ax, map-ont) (Li 2018). Small RNA-Seq reads were aligned to the genome using bowtie (options --mmap r) (Langmead and Salzberg 2012). Nanopore and small RNA RPKM were calculated using bamCoverage (options --bam --binSize 200 --ignoreDuplicates --normalizeUsing RPKM) (Ramirez *et al.* 2016). A general additive model was used to regress feature values across chromosome lengths (gam(<gene age or RPKM> ∼ s(Chrom.start, bs = “cs”, by = Chrom))), using the R package mgcv v.1.8–40. Gene age fits were plotted using the start position of each gene and RPKM fits were plotted using the start position of every 200 bp bin. Gene ages were randomly permuted 1,000 times, and the mean was plotted using the unchanged start position of each gene. A paired *t*-test grouped by chromosome was used to test the significance of the observed gene age distributions relative to random permutations.

### Sequence data retrieval, alignment, and phylogenetic analyses

A BlastP search was performed using the *Saccharomyces cerevisiae* protein sequence of FAS1 (CAA82025.1) and FAS2 (CAA97948.1) as the query sequence against fungal genomes. Sequences with >95% coverage and >40% identity were selected, and a total of 147 FAS homologs from 94 species were subjected to alignment and phylogenetic analysis. The Rozellomycota *Paramicrosporidium saccamoebae* FAS (PJF17744.1) was selected as an outgroup. Amino acids were aligned using MUSCLE5 (Edgar 2021). A maximum likelihood phylogenetic tree was inferred using RAxML-NG with 20 distinct starting trees using the best-fit model (LG + I + G4) selected by ModelTest-NG (Kozlov *et al.* 2019; Darriba *et al.* 2020). Bootstrapping converged after 100 replicates, branch support was assessed with Felsenstein's bootstraps, and bootstrapping convergence was tested using the autoMRE criterion within RAxML-NG. The ML tree was rooted using pxrr v1.2 within the phyx package (Brown *et al.* 2017).

## Results

### De novo assembly of the *R. irregularis* genome

Assembly using trimmed Nanopore reads resulted in 44 contigs that were polished using Illumina reads (Maeda *et al.* 2018). Two of these contigs were filtered out due to their size of <500 bp, resulting in a polished and filtered assembly of 42 contigs (Supplementary Fig. 1a). The assembly process produced a complete, circular mitochondrial genome of 70,793 bp, within the size range of other AM fungal mitochondrial genomes (Fig. 2a) (Lee and Young 2009; Nadimi *et al.* 2016). This mitochondrial genome was annotated using MitoHifi and contains sequences encoding transfer RNAs (tRNAS), ribosomal subunits, and genes typically identified on a fungal mitochondrial genome. Manual curation based on Hi-C read alignment to the nuclear genome assembly was used to assign the remaining 42 contigs to 32 chromosomal units (Fig. 2b). Prior to manual curation, contig N50 and L50 were 3,900,757 bp (∼3.9 Mb) and 15, respectively, rising to a scaffold N50 and L50 of 5,085,394 (∼5 Mb) and 13 post-curation (Table 1). Twenty-three of these scaffolds were complete and gapless chromosomes (Fig. 2c). Seventeen of the 32 chromosome-scale scaffolds of *R. irregularis* were produced telomere-to-telomere, with telomeric repeats of sequence TTAGGG_n_ identified at both 5′ and 3′ ends of the scaffolds, and an additional 14 containing 1 telomere (Fig. 2c). Average Illumina and Nanopore read coverage were highly uniform across all scaffolds, indicating that repetitive sequences are fully resolved (Supplementary Fig. 1b). The 32 chromosomes display extensive macro-synteny to a recent assembly of this species, except for stretches of chromosomes 1 and 5 (Supplementary Fig. 1c). This assembly suggests a misjoin in a previous assembly of this species, which would result in the potential overestimation of the number of *R. irregularis* chromosomes (Table 1). Research into the location of centromeric repeats of this symbiotic fungus may aid further analyses into chromosome number and structure of these organisms. The final haploid nuclear assembly following removal of the circular mitochondrial contig is 146,773,001 bp in size.

### Genome annotation using short- and long-read sequencing

**Fig. 3.**
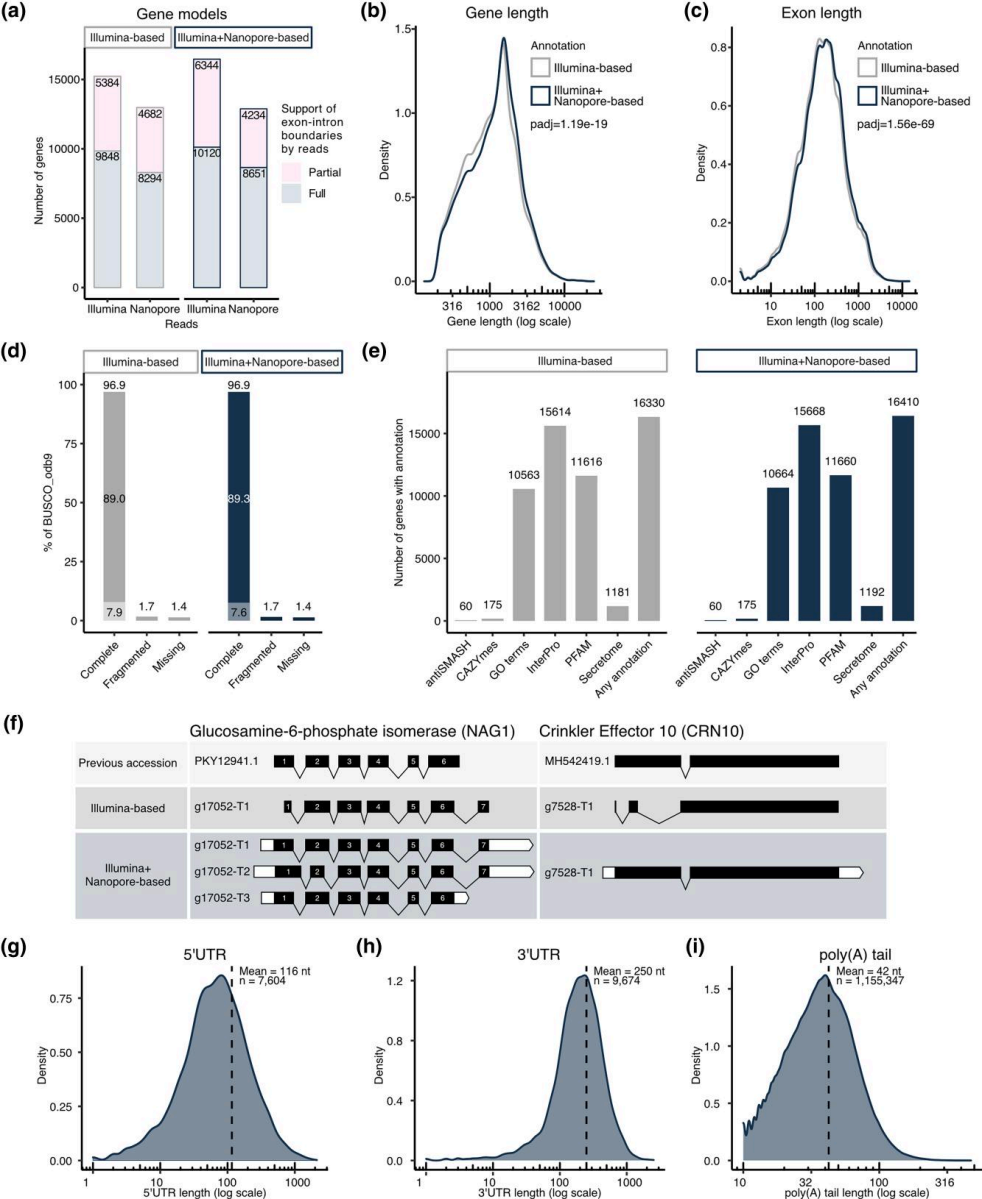
General features of revised gene models. a) Support of exon–intron boundaries of Illumina-based and Illumina + Nanopore-based gene annotations by Illumina and Nanopore RNA-Seq reads. The number of genes with all boundaries (full support) and partial boundaries (partial support) supported by experimental evidence is indicated. Exon-less genes are not displayed. Comparison of gene b) and exon c) length distribution between Illumina and Illumina + Nanopore-based gene annotations. The x-axes are on a log scale and a paired *t*-test was used to assess statistical significance. d) Comparison of BUSCO gene categories. The complete stack is split into single copy (top) and duplicated (bottom). e) Comparison of functional annotation of Illumina-based and Illumina + Nanopore-based gene models. f) Comparison of gene models revised using long-read data to previous annotations and Illumina-based gene models. Black boxes represent exons, lines are introns, and white boxes are UTRs. Length distribution of 5′UTRs g) and 3′UTRs h) of the Illumina + Nanopore gene models. i) Length distribution of poly(A) tails detected in spores. The *x*-axes are on a log scale.

Following modeling, curation, and masking of repetitive sequences and transposable elements, protein-coding genes were annotated using published Illumina RNA-Sequencing (RNA-Seq) reads from multiple life stages (Table 2). This Illumina-based gene annotation was manually curated to remove transposable elements, leaving 30,230 gene models (Illumina-based gene annotation). Gene models were then refined with long Nanopore RNA-Seq reads, improving the support of exon–intron boundaries by sequencing reads (Fig. 3a, Illumina + Nanopore-based annotation) and increasing gene and exon length (Fig. 3, b and c). Updated gene models were manually curated to remove transposable elements, resulting in a final annotation of 30,209 genes. This gene count is consistent with previous studies into genes encoded by AM fungal genomes (Morin *et al.* 2019). Long-read data did not change the overall BUSCO score (96.9%) but moved one duplicated BUSCO gene to the single-copy category (Fig. 3d). Functional annotation of gene models indicated that long reads increased the number of genes with assigned Gene Ontology (GO) terms (+101 genes), PFAM domains (+44 genes), InterPro domains (+54 genes), and secretion signals (+11 genes), while the number of biosynthetic genes and CAZymes remained constant (Fig. 3e). Refining gene models with long reads, therefore, resulted in more accurate gene models and a higher number of functionally annotated genes. Examples of updated gene models include glucosamine-6-phosphate isomerase (NAG1) and Crinkler effector 10 (CRN10), 2 genes thought to be involved in arbuscule development and function (Kobae *et al.* 2015; Voss *et al.* 2018). Compared to previous accessions, long-read data revealed 2 novel transcript isoforms of NAG1 that contain an additional exon (Fig. 3f; g17052-T1 and g17052-T2). A misannotated first intron of CRN10 was fixed, and the updated gene sequence is identical to the one described in Voss *et al.* (2018).

### Untranslated regions, poly(A) tails, and the poly(A) signal of *R. irregularis*

Long RNA-Seq reads provided evidence for untranslated region (UTR) prediction, polyadenylation site detection, and poly(A) tail length analyses; 5′UTR and 3′UTR length distributions have respective means of 116 and 250 nucleotides (nt) (Fig. 3, g and h), which are comparable to the fungal averages (134 and 237 nt, respectively) and within the known ranges of eukaryotic UTR lengths (100–200 nt 5′UTR, 200–1,000 nt 3′UTR) (Pesole *et al.* 2001; Mignone *et al.* 2002; Bruno *et al.* 2010; Lin and Li 2012). A MEME motif search in the 50 bp preceding the poly(A) tails of 242,742 unique poly(A) sites yielded one significantly enriched hexanucleotide motif, the canonical AAUAAA (Table 3, *E*-value 1.3e−24) (Bailey and Elkan 1994). This sequence accounts for 56.7% of detected poly(A) sites, indicating high sequence conservation to the mammalian polyadenylation signal, compared to yeast (13.2%), *Aspergillus oryzae* (6%), *Arabidopsis thaliana* (10%), and *Oryza sativa* (7%) (Table 3) (Graber *et al.* 1999; Loke *et al.* 2005; Shen *et al.* 2008; Tanaka *et al.* 2011). Additional derivatives such as AUUAAA and AAUAUA were also detected but were not significantly enriched. The distribution of poly(A) tail lengths in spore transcripts ranged from 10 to 473 nt, with a mean of 42 nt (Fig. 3i), which is comparable to the 50 nt average observed in *S. cerevisiae* using similar methods (Tudek *et al.* 2021).

**Table 3.** *Rhizophagus irregularis* polyadenylation signal(s).

Sequence	Number	Percent
AAUAAA	137,539	56.7
CAUAAA	212	0.1
GAUAAA	328	0.1
UAUAAA	4581	1.9
ACUAAA	169	0.1
AGUAAA	475	0.2
AUUAAA	28,045	11.6
AACAAA	431	0.2
AAGAAA	481	0.2
AAUACA	429	0.2
AAUAGA	266	0.1
AAUAUA	11,489	4.7
AAUAAC	196	0.1
AAUAAG	117	0.0
AAUAAU	1876	0.8
CAUGAA	26	0.0
GAUGAA	96	0.0
UAUGAA	336	0.1

### A burst of gene novelty with the emergence of Glomeromycotina fungi

**Fig. 4.**
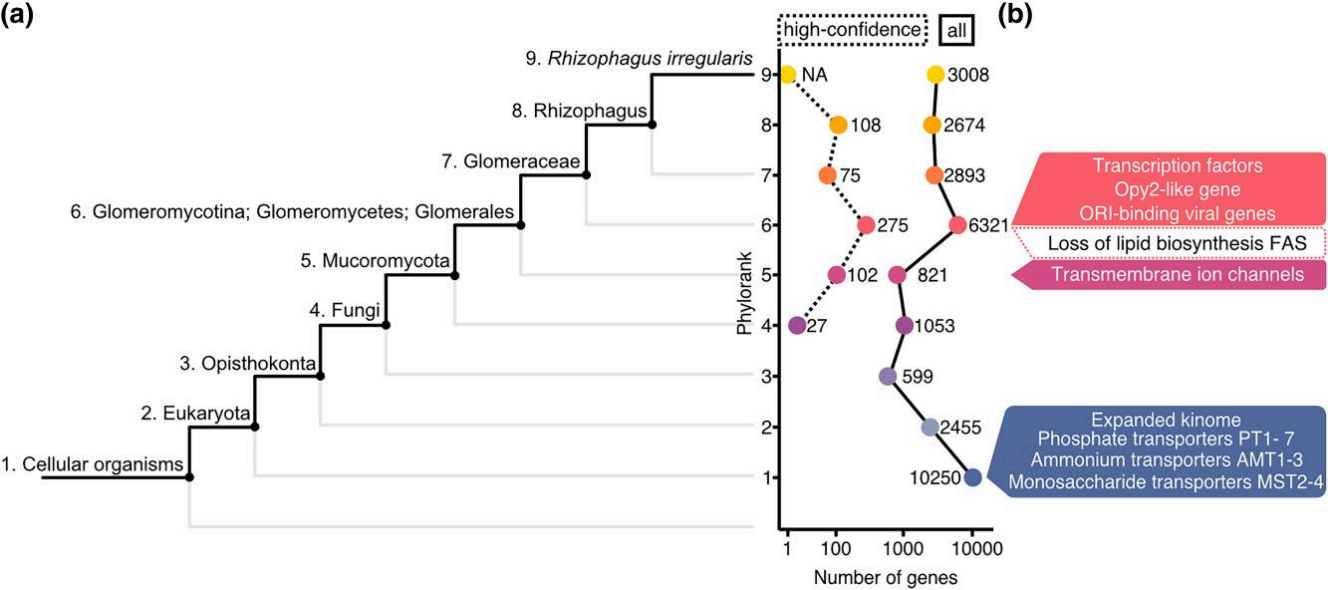
Phylostratigraphy analysis of *Rhizophagus irregularis* genes. a) Left panel: *R. irregularis* lineage. Right panel: number of genes at each phylorank before (full line) and after (dashed line) accounting for homology detection failure (HDF). The dashed line represents genes with high confidence phyloranks that could not be explained by HDF. b) Model of gene birth and gene loss in the *R. irregularis* lineage.

A tree of life scale comparative genomics analysis was used to estimate the evolutionary ages of *R. irregularis* genes, tracing gene birth events to the last universal common ancestor (Barrera-Redondo *et al.* 2023). This analysis suggests that 34% (*n* = 10,250) of *R. irregularis* genes have homologs across taxonomic levels and date back to the origin of cellular organisms (Fig. 4a, all genes). This most ancient phylorank (phylorank 1) is enriched for basic cellular functions and primary metabolic processes such as transcription, translation, and regulation of cell cycle (Supplementary Table 1), which are expected to be conserved across the tree of life. Notably, 2,373 out of 2,533 members of *R. irregularis*’ expanded kinase gene repertoire are found at phylorank 1, consistent with protein phosphorylation as a fundamental mechanism of cell signaling (Supplementary Tables 1 and 2) (Kwon *et al.* 2019). All phosphate transporters (PT1 to PT7), ammonium transporters (AMT1, AMT2, AMT3), and monosaccharide transporters (MST2, MST3, MST4) are found at phylorank 1 (Table 4). As may be expected, this analysis suggests that phosphate, nitrogen, and carbohydrate efflux and homeostasis are ancestral molecular functions that emerged long before AM fungi. Our analysis revealed comparable numbers of highly conserved genes in the Glomeromycotina fungi *Gigaspora margarita* (40%, *n* = 11,731) and *Geosiphon pyriformis* (46%, *n* = 6,875) (Supplementary Fig. 2a, phylorank 1). The Glomeromycotina, Mucoromycotina, and Mortierellomycotina species analyzed here share similar gene age distributions until the emergence of the Mucoromycota, where each lineage displays their independent historical patterns of gene emergence (Supplementary Fig. 2a, phyloranks 1 to 5). A peak of gene birth events at the Glomeromycotina phylorank indicates that the emergence of this fungal lineage is marked by a burst of lineage-restricted evolutionary novelties (Fig. 4a, phylorank 6, all genes). One caveat of phylostratigraphy is that gene age is often underestimated because of the inability of pairwise aligners to trace back homologs in outgroups that are too evolutionarily distant. Robust assessment of gene birth events, therefore, relies on testing the null hypothesis of homology detection failure (HDF) in order to achieve high-confidence predictions (Barrera-Redondo *et al.* 2023). A more stringent analysis taking into account HDF of recently evolved genes confirmed the burst of gene birth in Glomeromycotina (Fig. 4a, phylorank 6, high confidence). Confidently ranked genes born in the Glomeromycotina include an HTH APSES-type transcription factor (g4815), a Zn(2)-C6 fungal-type transcription factor (g25112), an Opy2-like membrane anchor protein (g2640), 2 uncharacterized Crinkler-type effectors (g11050, g27662), a Complex 1 LYR protein (g6617), and many F-box and Leucine repeat genes (Fig. 4b, and Supplementary Tables 2 and 3). Two GO terms related to replication were enriched at the high confidence phylorank 6 and are linked to genes of potential viral origin (Fig. 4b and Table 5). These genes have putative replication-origin-binding domains (InterPro domain IPR003450) and were most likely acquired through horizontal transfer in the common ancestor of Glomeromycotina and subsequently inherited vertically throughout the whole lineage. Genes born at the emergence of Glomeromycotina may encode functions that were crucial for their evolutionary success and diversification, such as developmental innovation for symbiosis or obligate biotrophy.

**Table 4.** Nutrient transporter genes at phylorank 1.

Function	Gene Name	Gene ID
Phosphate transporter	PT1	g11592
	PT2	g7615
	PT3	g111
	PT4	g31083
	PT5	g18438
	PT6	g27858
	PT7	g19437
Ammonium transporter	AMT1	g16666
	AMT2	g1222
	AMT3	g18142
Monosaccharide transporter	MST2	g24501
	MST3	g19549
	MST4	g26862

**Table 5.** GO terms enriched in genes with high confidence phyloranks.

Phylorank	Enriched GO description	Enriched GO term	*P*_adj_	Gene ID
8	NA	NA	NA	NA
7	NA	NA	NA	NA
6	DNA replication origin binding	GO:0003688	7.4E-03	g15381 g20925 g2621
6	Sequence-specific double-stranded DNA binding	GO:1990837	3.6E-02	
5	NA	NA	NA	NA

Although the ages of most genes at the Mucoromycota phylorank may be underestimated without accounting for HDF, general shifts in protein sequence space can still be captured (Domazet-Lošo *et al.* 2022). GO term enrichment analyses were performed to investigate molecular functions that are ancestral to Glomeromycotina. GO terms related to ion transport, transmembrane transporter activity, and membrane components are significantly enriched at the Mucoromycota phylorank (Supplementary Table 1, phylorank 5). The genes underlying this enrichment mainly consisted of a group of 32 transient receptor channel subfamily V-like genes with predicted permeability to Ca^2+^ (Fig. 4b and Table 6) (Nilius and Szallasi 2014). Innovation in transmembrane ion transport, therefore, precedes Glomeromycotina and may be a feature that marked the evolutionary transition of Mucoromycota fungi.

**Table 6.** Genes with enriched membrane and ion transport GO terms at Mucoromycota phylorank 5.

GO term ID	Description	*P*.val	False Discovery Rate	Genes
GO:0005216	Ion channel activity	4.4E-35	4.4E-35	g12620, g14470, g14472, g14478, g17590, g17616, g17700, g17949, g22276, g22280, g22289, g22304, g22306, g22308, g22310, g22315, g22331, g22332, g22338, g22346, g22348, g22357, g22367, g22372, g22476, g22485, g22497, g25077, g25094, g31340, g6647, g6926
GO:0022803	Passive transmembrane transporter activity	1.5E-33	1.5E-33	g12620, g14470, g14472, g14478, g17590, g17616, g17700, g17949, g22276, g22280, g22289, g22304, g22306, g22308, g22310, g22315, g22331, g22332, g22338, g22346, g22348, g22357, g22367, g22372, g22476, g22485, g22497, g25077, g25094, g31340, g6647, g6926
GO:0015267	Channel activity	1.5E-33	1.5E-33	g12620, g14470, g14472, g14478, g17590, g17616, g17700, g17949, g22276, g22280, g22289, g22304, g22306, g22308, g22310, g22315, g22331, g22332, g22338, g22346, g22348, g22357, g22367, g22372, g22476, g22485, g22497, g25077, g25094, g31340, g6647, g6926
GO:0006811	Ion transport	8.5E-32	8.5E-32	g12620, g14470, g14472, g14478, g17590, g17616, g17700, g17949, g22276, g22280, g22289, g22304, g22306, g22308, g22310, g22315, g22331, g22332, g22338, g22346, g22348, g22357, g22367, g22372, g22476, g22485, g22497, g25077, g25094, g31340, g6647, g6926
GO:0015318	Inorganic molecular entity transmembrane transporter activity	1.4E-28	1.4E-28	g12620, g14470, g14472, g14478, g17590, g17616, g17700, g17949, g22276, g22280, g22289, g22304, g22306, g22308, g22310, g22315, g22331, g22332, g22338, g22346, g22348, g22357, g22367, g22372, g22476, g22485, g22497, g25077, g25094, g31340, g6647, g6926
GO:0015075	Ion transmembrane transporter activity	1.4E-26	1.4E-26	g12620, g14470, g14472, g14478, g17590, g17616, g17700, g17949, g22276, g22280, g22289, g22304, g22306, g22308, g22310, g22315, g22331, g22332, g22338, g22346, g22348, g22357, g22367, g22372, g22476, g22485, g22497, g25077, g25094, g31340, g6647, g6926
GO:0031224	Intrinsic component of membrane	3.9E-22	3.9E-22	g12620, g14470, g14472, g14478, g17590, g17616, g17700, g17949, g22276, g22280, g22289, g22304, g22306, g22308, g22310, g22315, g22331, g22332, g22338, g22346, g22348, g22357, g22367, g22372, g22476, g22485, g22497, g25077, g25094, g28535, g31340, g6647, g6778, g6926, g7404, g7406, g7408, g9272, g9509
GO:0016021	Integral component of membrane	3.9E-22	3.9E-22	g12620, g14470, g14472, g14478, g17590, g17616, g17700, g17949, g22276, g22280, g22289, g22304, g22306, g22308, g22310, g22315, g22331, g22332, g22338, g22346, g22348, g22357, g22367, g22372, g22476, g22485, g22497, g25077, g25094, g28535, g31340, g6647, g6778, g6926, g7404, g7406, g7408, g9272, g9509
GO:0022857	Transmembrane transporter activity	9.8E-20	9.8E-20	g12620, g14470, g14472, g14478, g17590, g17616, g17700, g17949, g22276, g22280, g22289, g22304, g22306, g22308, g22310, g22315, g22331, g22332, g22338, g22346, g22348, g22357, g22367, g22372, g22476, g22485, g22497, g25077, g25094, g31340, g6647, g6926
GO:0005215	Transporter activity	6.6E-19	6.6E-19	g12620, g14470, g14472, g14478, g17590, g17616, g17700, g17949, g22276, g22280, g22289, g22304, g22306, g22308, g22310, g22315, g22331, g22332, g22338, g22346, g22348, g22357, g22367, g22372, g22476, g22485, g22497, g25077, g25094, g31340, g6647, g6926
GO:0016020	Membrane	6.6E-14	6.6E-14	g12620, g14470, g14472, g14478, g17590, g17616, g17700, g17949, g22276, g22280, g22289, g22304, g22306, g22308, g22310, g22315, g22331, g22332, g22338, g22346, g22348, g22357, g22367, g22372, g22476, g22485, g22497, g25077, g25094, g28535, g31340, g6647, g6778, g6926, g7404, g7406, g7408, g9272, g9509
GO:0006810	Transport	9.6E-14	9.6E-14	g12620, g14470, g14472, g14478, g16535, g17590, g17616, g17700, g17949, g22276, g22280, g22289, g22304, g22306, g22308, g22310, g22315, g22331, g22332, g22338, g22346, g22348, g22357, g22367, g22372, g22476, g22485, g22497, g25077, g25094, g31340, g6647, g6926
GO:0055085	Transmembrane transport	4.4E-02	4.4E-02	g14478, g22276, g22280, g22306, g22308, g22315, g22331, g22338, g22346, g22485, g25077

### Fatty acid auxotrophy resulted from the loss of a single fatty acid synthase gene in the Glomeromycotina ancestor

**Fig. 5.**
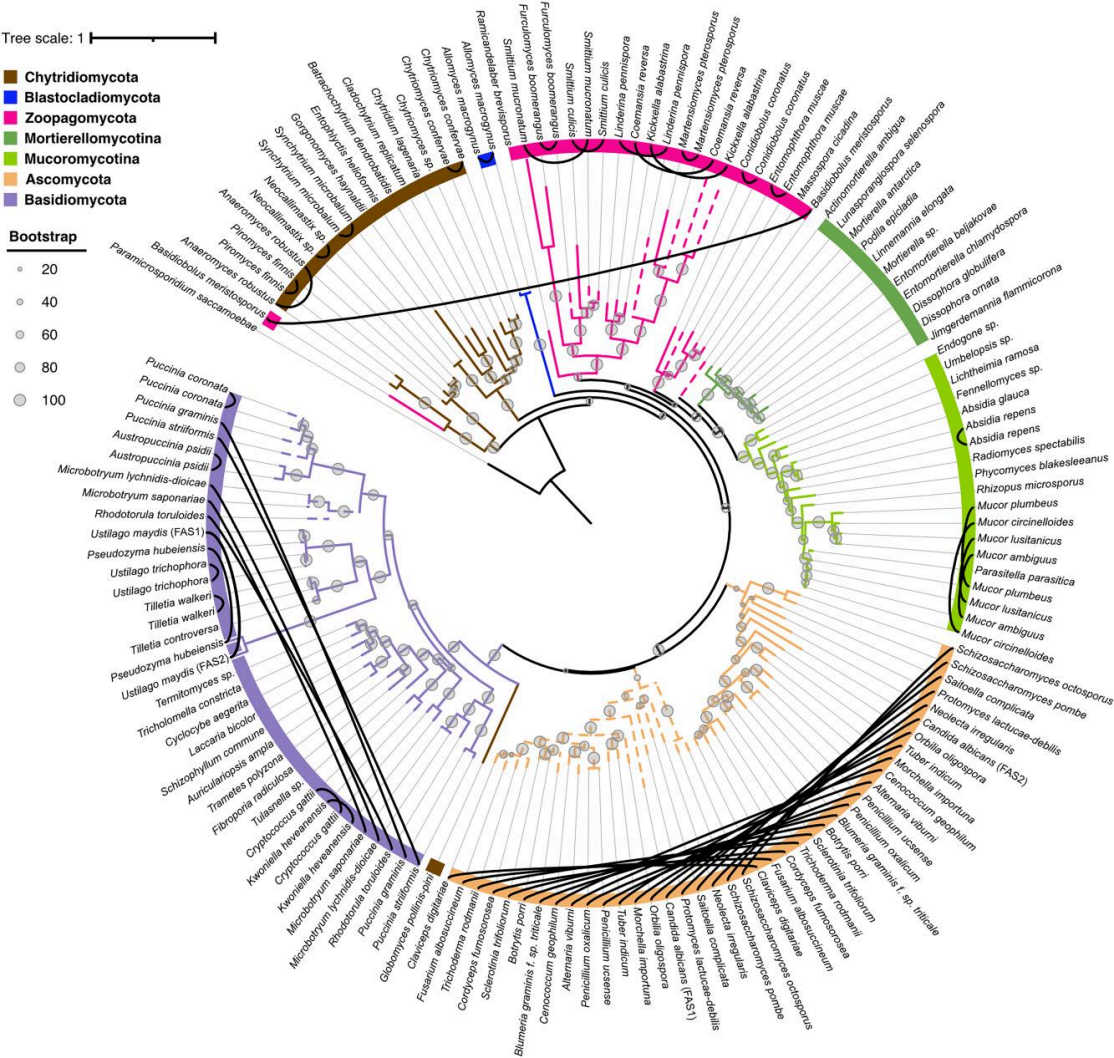
The fungal de novo FAS gene family. A rooted phylogenetic tree of 147 fungal FAS protein models inferred from maximum likelihood (RAxML). Main phyla and Mucoromycota subphyla are highlighted by colored branches and boxes. Black lines link within-species paralogs, and paralog branches are dashed. Bootstrap support values are shown at the nodes.

In animals, the entire pathway of de novo fatty acid synthesis relies on a single cytosolic enzyme, the fatty acid synthase (FAS). The evolution of FAS genes is more complex in fungi, which can encode the enzymatic domains of FAS on a single gene or on 2 genes (Bukhari *et al.* 2014). It has long been known that no FAS gene can be recovered from genomes of AM fungi (Wewer *et al.* 2014; Malar *et al.* 2021) and that this loss causes dependence on host-derived lipids (Trepanier *et al.* 2005; Bravo *et al.* 2017; Jiang *et al.* 2017; Keymer *et al.* 2017; Luginbuehl *et al.* 2017). However, it is still unclear whether AM fungi have lost a single FAS gene or 2 FAS genes (in a possibly gradual fashion). A protein search of fungal FAS sequences revealed that most early-diverging fungi have one multi-domain FAS gene (Fig. 5; Chytridiomycota, Zoopagomycota, Mucoromycota). Instances of early-diverging species with 2 FAS genes are explained by duplications and while some paralogs appear to have significantly diverged (e.g. *Basidiobolus meristosporus*), most possess all core protein domains (Supplementary Fig. 3). Mucoromycota FAS genes are phylogenetically clustered and, although recent duplications exist (e.g. in Mucor sp.), most species have one FAS (Fig. 5). The ancestor of Glomeromycotina likely had one FAS gene, and lipid auxotrophy in AMF resulted from loss of this single-gene FAS, and not multiple, gradual losses. Only at a later stage in Dikarya evolution, and more prominently in Ascomycota, was the multi-domain FAS gene split into 2 genes with different protein domains, FAS1 and FAS2 (β- and α-subunits, respectively) (Fig. 5, and Supplementary Fig. 3). In Ascomycota, the phylogenetic topology and branch lengths of fissioned subunits are remarkably similar (Fig. 5) and likely reflect an evolutionary requirement for maintaining physical interactions and orchestrating molecular assembly into a 2.6 megadalton barrel-shaped complex (Jenni *et al.* 2007; Fischer *et al.* 2015, 2020). FAS paralogs in early-diverging fungi may operate as homomers or heteromers as a result of a gene fusion in the common ancestor of fungi and animals (Supplementary Fig. 3b) (Marsh *et al.* 2013).

### Variation in gene age along chromosomes reveals that evolutionarily young loci produce abundant small RNAs

**Fig. 6.**
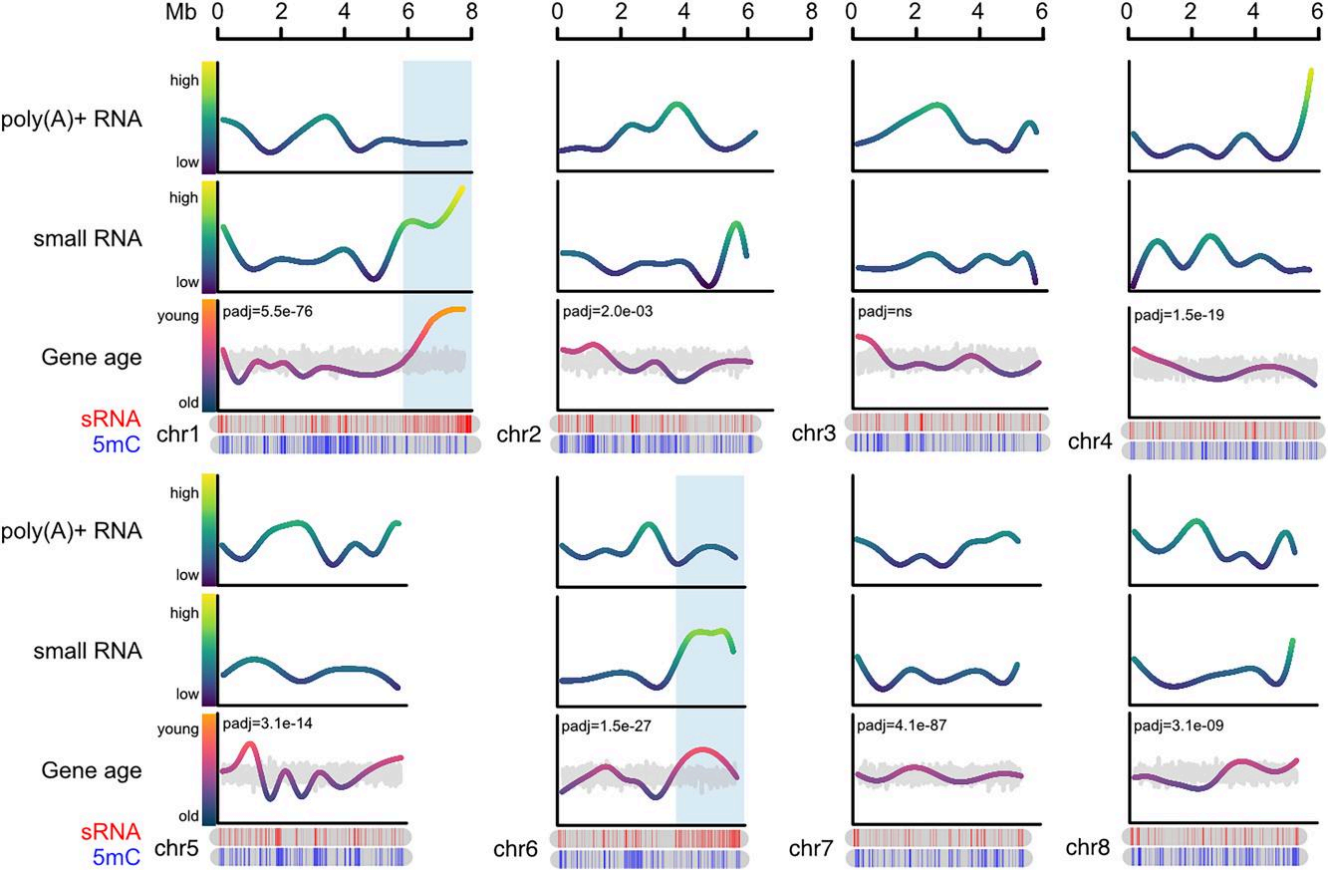
Genome-wide patterns of gene age and expression. Per-chromosome genomic distribution of Nanopore polyA+ RNA-Seq (top line graph, long RNA), expression of small non-coding RNAs (RPKM, second line graph, sRNA), gene age (third line graph, old to young corresponding to phyloranks 1 to 9), small RNA loci (top ideogram, sRNA), and highly methylated CGs (bottom ideogram, 5 mC values > 80% are shown, 5 mC). Color gradients of line graphs match the y-axis scales. Grey line graphs overlapping with gene ages represent the mean of 1,000 random permutations of gene ages, and a paired *t*-test (grouped by chromosome) was used to test the significance of observed gene age distributions relative to random permutations *P*_adj_). Values used for nonparametric linear regressions of long and small RNA expression are normalized RPKM calculated in 200 bp bins. Gene ages are regressed and plotted following chromosomal gene distribution (not binned). Blue-shaded regions highlight 2 loci containing the youngest genes and the highest concentration of highly expressed small RNA loci. The first 8 chromosomes are shown here, and chromosomes 9–32 are shown in Supplementary Fig. 4a.

To investigate genome-wide patterns of feature distribution, a series of datasets were mapped to *R. irregularis* chromosomal scaffolds using nonparametric linear regressions. Normalized reads per kilobase per million reads mapped (RPKM) of Nanopore RNA-Seq (full-length, poly(A)-selected) and small RNA-Seq (∼24 nt long) were reported in 200 bp genomic bins, and gene ages (pre-HDF test) were measured across the chromosomal length (Fig. 6 and Supplementary Fig. 4a). An uneven distribution of gene age was observed for all chromosomes, distinguishing regions enriched with evolutionarily ancient genes (low phyloranks) from regions with evolutionarily young genes (high phyloranks) (Fig. 6 and Supplementary Fig. 4a). Genomic regions with evolutionarily ancient genes tend to have high poly(A)+ RNA and low small RNA expression levels, and these patterns are reversed in regions with evolutionarily young genes (Fig. 6 and Supplementary Fig. 4, a–e). However, a small number of genes produce small RNAs (Dallaire *et al.* 2021), and when examined at the scale of individual genes, per-gene small RNA expression levels did not correlate with gene age (Supplementary Fig. 4d). This led to the conclusion that sequence surrounding young genes, rather than young genes themselves, drive the observed pattern of small RNA expression. Two particular loci of ∼2 Mbps in length were identified (Fig. 6, regions shaded in blue) that collectively contain evolutionarily young coding regions and the most abundant concentration of highly expressed small RNA loci. These data suggest that the genome of *R. irregularis* presents highly transcribed regions harboring highly conserved genes, and lesser transcribed, small RNA-producing regions with evolutionarily younger genes.

## Discussion

The number of fungal species with highly contiguous, long-read, and chromosome-scale assemblies lags behind that of animals and plants (Marks *et al.* 2021; Rhie *et al.* 2021). This work presents a chromosome-scale assembly of the symbiotic fungus *R. irregularis*, isolate DAOM197198, the model species for molecular research into AM fungi. This assembly of 32 chromosomal scaffolds is highly contiguous, with only 10 gaps and a contig N50 of 3.9 Mb. Nuclear chromosomes display a very high synteny with those of a previous assembly of *R. irregularis* DAOM197198 (Supplementary Fig. 1c) (Yildirir *et al.* 2022), though this assembly assigns sequence to 32 chromosomal scaffolds, in contrast to the 33 chromosomal scaffolds previously presented. A complete, gapless, circular mitochondrial genome of 70,793 bp was also assembled, a size consistent with a previous assembly of the *R. irregularis* mitochondria (Lee and Young 2009). This novel assembly, alongside a high-quality genome annotation of *R. irregularis*, consisting of gene models with corrected structures, splice junctions, and untranslated regions, will further aid research into *R. irregularis* and AM fungal biology, as well as comparative genomics approaches.

This highly contiguous genome assembly enabled an analysis of chromosomal distributions of *R. irregularis* genomic features and gene and small RNA expression. This supports a previous observation of functional and evolutionary genome compartmentalization in *R. irregularis* (Yildirir *et al.* 2022) and builds on this work by showing that chromosomes contain highly expressed regions with highly conserved genes, and lowly expressed regions hosting more recently evolved genes. This is reminiscent of the 2-speed genome model, which has been described in filamentous phytopathogens (Torres *et al.* 2020) and proposed to exist in AM fungi (Reinhardt *et al.* 2021; Yildirir *et al.* 2022). According to this model, fast-evolving virulence-associated genes are compartmentalized into repeat-rich genomic regions or accessory chromosomes that are depleted of conserved housekeeping genes. In the plant pathogenic fungus *Sclerotinia sclerotiorum*, small RNAs originate from transposable elements in polymorphic genome compartments (Derbyshire *et al.* 2019). In *R. irregularis*, quantitative evidence for differential evolutionary speed and sequence variation in genomics compartments is lacking. Nevertheless, evolutionary patterns of genomic architecture can be observed, as well as small RNA production in regions with evolutionarily young coding spaces (Fig. 6). Evolutionary and functional compartmentalization of genes is likely not limited to species with pathogenic lifestyles, and future work will further elucidate its role as a general evolutionary feature. Analysis of genome-wide patterns of small RNA expression may point to loci that encode the basis for lineage-specific adaptations and diversification in AM fungi and may facilitate studies into adaptive structural and sequence variation.

With the increasing number of reference genomes available for Earth's biodiversity (Lewin *et al.* 2018) and the development of efficient algorithms for sequence analysis (Buchfink *et al.* 2021; Jumper *et al.* 2021), characterization of genes and genomes can harness comparisons at tree-of-life scale. This study used a phylostratigraphic gene age inference tool that performs alignments against the entire NCBI non-redundant protein database to trace back the emergence of *R. irregularis* genes (Barrera-Redondo *et al.* 2023). Genetic machinery for phosphate, ammonium, monosaccharide transport, ion transmembrane transport, and a group of transmembrane ion channels were found to have evolved at or before the Mucoromycota phylorank, thereby predating the emergence of Glomeromycotina. The evolution of ion transporters in AMF's ancestors may have been crucial for maintaining intracellular ion balance in organisms that harvest high levels of negatively charged phosphate from the soil. A similar phenomenon was observed in the genomes of saprotrophic fungi, which encode the symbiosis toolkit of their successor ectomycorrhizal species (Hess *et al.* 2018; Miyauchi *et al.* 2020). Similarly in plants, the genetic basis for symbiont perception, nodule organogenesis, and nitrogen-fixation genes already existed in the common ancestor of nitrogen-fixing legumes and diversified in downstream nitrogen-fixing lineages (Libourel *et al.* 2022). Such macroevolutionary transitions punctuate the eukaryotic tree of life, where the acquisition of new molecular functions accompanies major evolutionary and ecological transitions but precedes divergence and lifestyle specialization in downstream lineages (Domazet-Lošo *et al.* 2022; Ocana-Pallares *et al.* 2022).

The detection of a gene birth event accompanying the emergence of Glomeromycotina highlights the existence of previously undescribed lineage-restricted innovation. Gene birth events associate with the emergence of ectomycorrhizal lifestyles (Hess *et al.* 2018; Miyauchi *et al.* 2020) and of rhizoid and root development in land plants (Barrera-Redondo *et al.* 2023). While the loss of the gene encoding de novo FAS activity likely played a major role in creating dependence to externally supplied carbon (Trepanier *et al.* 2005; Bravo *et al.* 2017; Jiang *et al.* 2017; Keymer *et al.* 2017; Luginbuehl *et al.* 2017; Malar *et al.* 2021), the birth of lineage-restricted genes such as the transcription factors identified here may also underlie an evolutionary transition in the Glomeromycotina subphylum.

## Supplementary Material

jkad077_Supplementary_Data

## Data Availability

DNA and RNA sequencing datasets, genome assembly, and annotations generated are available at PRJNA885267. Previously published datasets used are GSE172187 and PRJNA722386 from Dallaire *et al.* (2021), PRJNA748024 from Yildirir *et al.* (2022), and DRA004835 from Maeda *et al.* (2018). Code and Supplemental Files are available at https://github.com/bethanmanley and https://doi.org/10.5281/zenodo.7713976. Supplemental material available at G3 online.
