# Supplementary material for: A highly contiguous genome assembly reveals sources of genomic novelty in the symbiotic fungus Rhizophagus irregularis
Source: G3 (Bethesda). 2023 Mar 31;13(6):jkad077. doi: 10.1093/g3journal/jkad077 (PMC10234402; doi:10.1093/g3journal/jkad077)
Supplement: jkad077_Supplementary_Data [file jkad077_supplementary_data.docx]

**Supplemental Information**

Supplemental Data and Files are available at BioProject PRJNA885267, <https://github.com/bethanmanley>, <https://doi.org/10.5281/zenodo.7713976>, and https://zenodo.org/record/7126157#.Y2-L2i-l3Aa.

**Supplemental Files**

Raw Nanopore DNA and RNA reads (BioProject PRJNA885267)

Rhizophagus_irregularis_DAOM197198_Nanopore_DNASeq_Run1.fastq.gz

Rhizophagus_irregularis_DAOM197198_Nanopore_DNASeq_Run2.fastq.gz

Rhizophagus_irregularis_DAOM197198_Nanopore_DNASeq_Run3.fastq.gz

Rhizophagus_irregularis_DAOM197198_Nanopore_RNASeq.fastq.gz

Nuclear genome assembly, masked and unmasked

Rhizophagus_irregularis_DAOM197198_assembly.fasta

Illumina and Illumina+Nanopore genes, CDS, mRNA, protein, and functional annotations

Rhizophagus_irregularis_DAOM197198_Illumina+ONT_curated.gff3

Rhizophagus_irregularis_DAOM197198_Illumina_curated.gff3

Rhizophagus_irregularis_DAOM197198_annotations_Illumina+ONT.txt

Rhizophagus_irregularis_DAOM197198_annotations_Illumina.txt

Rhizophagus_irregularis_DAOM197198_cds-transcripts_Illumina+ONT_curated.fa

Rhizophagus_irregularis_DAOM197198_cds-transcripts_Illumina_curated.fa

Rhizophagus_irregularis_DAOM197198_mrna-transcripts_Illumina+ONT_curated.fa

Rhizophagus_irregularis_DAOM197198_mrna-transcripts_Illumina_curated.fa

Rhizophagus_irregularis_DAOM197198_proteins_Illumina+ONT_curated.fa

Rhizophagus_irregularis_DAOM197198_proteins_Illumina_curated.fa

GO terms for g:Profiler
Rhizophagus_irregularis_DAOM197198_Illumina+ONT_GOterms.gmt

Repetitive and transposable element annotation

Rhizophagus_irregularis_DAOM197198_curatedrepeatlibrary.fasta

Rhizophagus_irregularis_DAOM197198_repeatmasker.out

Rhizophagus_irregularis_DAOM197198_repeats.gff3

DNA methylome (spores)

Rhizophagus_irregularis_DAOM197198_mCG_mods_frequency.tsv

Poly(A) signal and tail sequences

Rhizophagus_irregularis_DAOM197198_pasa_polyAsite_analysis.out

Rhizophagus_irregularis_DAOM197198_pasa_polyAsites.fasta

Small RNA annotation

Rhizophagus_irregularis_DAOM197198_sRNA.gff3

Rhizophagus_irregularis_DAOM197198_sRNA.tsv

Mitochondrial genome assembly and annotation

Rhizophagus_irregularis_DAOM197198_mtDNA.fasta

Rhizophagus_irregularis_DAOM197198_mtDNA.gff

*R. irregularis* phylostratigraphy

Rhizophagus_irregularis_DAOM197198_1432141_phyloranks.tsv

Rhizophagus_irregularis_DAOM197198_1432141_high-confidence_phyloranks.tsv

Mucoromycota fungi phylostratigraphy

Disdec1_101101_phyloranks.tsv

Geopyr1_50956_phyloranks.tsv

Gigmar1_4874_phyloranks.tsv

Morel2_1314771_phyloranks.tsv

Phybl2_4837_phyloranks.tsv

Radspe1_64574_phyloranks.tsv

Fatty acid synthase phylogeny

FAS_genes_muscle5_msa.fa (alignments)

FAS_genes.raxml.support (ML tree)

**Supplemental Figures**

**Figure S1.** Assembly graph, coverage and macro-synteny analysis

A) Raw assembly graph following Shasta assembly. Ambiguities in the graph structure are represented by edges (fine black lines). Contigs indicated with a star (*) have lengths of 212bp and 164bp and were removed from the assembly due to their size. The circular mitochondrial contig is displayed as the final contig. B) A cumulative distribution indicating the proportion of total bases that were covered by Nanopore (top) and Illumina (bottom) reads, pre-curation. Each line represents an assembled contig. C) Whole-genome pairwise alignment between the current assembly and the Yildirir et al., 2022 assembly. Blue dots represent unique alignments on the positive strand of both assemblies, green dots represent unique alignments on the negative strand, and orange dots represent non-unique alignments (repetitive).

**Figure S2.** Distribution of gene age in Mucoromycota fungi

A) Gene age assignment for Glomeromycotina (*Geosiphon pyriformis*, *Gigaspora margarita* and *Rhizophagus irregularis*), Mortierellomycotina (*Dissophora decumbens* and *Mortierella elongata*) and Mucoromycotina (*Radiomyces spectabilis* and *Phycomyces blakesleeanus*) species. Log-transformed number of genes in each phylorank is displayed. Grey shaded areas highlight phyloranks that were collapsed due to insufficient genomic data, which can result in overestimation of gene birth. B), C) and D) show possible phylogenetic placement of the three subphyla.

**Figure S3.** Protein domain architecture of the fungal FAS family

A) mv-plot representation of PFAM domain conservation among FAS proteins from each phylum (Schlapfer et al. 2021). A representative model of domain architecture is shown under each plot. B) Model of FAS gene fusion and fissions reported across the Tree of Life. In bacteria and plants, FAS components are encoded by multiple genes. Plant FAS genes are shared between the plastid and mitochondria (Guan et al. 2020). FAS genes were fused into one multi-domain gene in the common ancestor of animals and fungi. Early-diverging fungi have one FAS gene, which was lost in Glomeromycotina and later split in two parts in all Ascomycota and in some of the Basidiomycota species analysed. Gene splitting was reported at two different positions (Jenni et al. 2007), shown as dashed white lines.

**Figure S4.** Correlation of genomic feature distributions

A) Genome-wide patterns of gene age and expression for chromosomes 9 to 32. Refer to Figure 6 legend for detailed description. B) Distribution of raw values of normalised Nanopore RNA RPKM, in 200bp bins, across phyloranks. C) Scatter plot showing linear regression values of normalised Nanopore RNA RPKM and phylorank values, for a subsample of 200bp genomic bins. D) Distribution of raw values of normalised small RNA RPKM measured at gene level, across phyloranks. E) Scatter plot showing linear regression values of normalised genome-wide small RNA RPKM and phylorank values, for a subsample of 200bp genomic bins.

**Supplemental Tables**

**Table S1.** GO terms enriched in all genes

| **Phylorank** | **Enriched GO description** | **Enriched GO term** | **padj** |
| --- | --- | --- | --- |
| 9 | Protein binding | GO:0005515 | 1.70E-02 |
| 8 | Protein binding | GO:0005515 | 3.40E-32 |
| 8 | Binding | GO:0005488 | 2.00E-24 |
| 8 | Molecular function | GO:0003674 | 4.90E-04 |
| 8 | Outer mitochondrial membrane organization | GO:0007008 | 5.00E-02 |
| 8 | Protein insertion into mitochondrial outer membrane | GO:0045040 | 5.00E-02 |
| 7 | Protein binding | GO:0005515 | 2.60E-48 |
| 7 | Binding | GO:0005488 | 3.90E-35 |
| 7 | Molecular function | GO:0003674 | 2.70E-04 |
| 7 | RNA binding | GO:0003723 | 4.80E-02 |
| 6 | Protein binding | GO:0005515 | 8.00E-89 |
| 6 | Binding | GO:0005488 | 1.20E-65 |
| 6 | Zinc ion binding | GO:0008270 | 8.70E-15 |
| 6 | Molecular function | GO:0003674 | 3.20E-07 |
| 6 | Cation binding | GO:0043169 | 3.90E-06 |
| 6 | Transition metal ion binding | GO:0046914 | 7.60E-06 |
| 6 | Metal ion binding | GO:0046872 | 2.30E-05 |
| 5 | ion channel activity | GO:0005216 | 4.40E-35 |
| 5 | passive transmembrane transporter activity | GO:0022803 | 1.50E-33 |
| 5 | channel activity | GO:0015267 | 1.50E-33 |
| 5 | ion transport | GO:0006811 | 8.50E-32 |
| 5 | inorganic molecular entity transmembrane transporter activity | GO:0015318 | 1.40E-28 |
| 5 | ion transmembrane transporter activity | GO:0015075 | 1.40E-26 |
| 5 | intrinsic component of membrane | GO:0031224 | 3.90E-22 |
| 5 | integral component of membrane | GO:0016021 | 3.90E-22 |
| 5 | transmembrane transporter activity | GO:0022857 | 9.80E-20 |
| 5 | transporter activity | GO:0005215 | 6.60E-19 |
| 5 | establishment of localization | GO:0051234 | 5.50E-14 |
| 5 | membrane | GO:0016020 | 6.60E-14 |
| 5 | transport | GO:0006810 | 9.60E-14 |
| 5 | RNA binding | GO:0003723 | 2.70E-08 |
| 5 | cellular anatomical entity | GO:0110165 | 2.90E-07 |
| 5 | cellular component | GO:0005575 | 2.00E-06 |
| 5 | nucleic acid binding | GO:0003676 | 3.50E-06 |
| 5 | heterocyclic compound binding | GO:1901363 | 5.40E-05 |
| 5 | organic cyclic compound binding | GO:0097159 | 5.50E-05 |
| 5 | G protein-coupled receptor activity | GO:0004930 | 2.30E-03 |
| 5 | G protein-coupled receptor signaling pathway | GO:0007186 | 2.00E-02 |
| 5 | transmembrane transport | GO:0055085 | 4.40E-02 |
| 5 | endoplasmic reticulum inheritance | GO:0048309 | 5.00E-02 |
| 5 | organelle inheritance | GO:0048308 | 5.00E-02 |
| 4 | kinetochore | GO:0000776 | 3.20E-09 |
| 4 | nuclear protein-containing complex | GO:0140513 | 4.80E-09 |
| 4 | transcription regulator activity | GO:0140110 | 3.30E-08 |
| 4 | transcription regulator activity | GO:0140110 | 3.30E-08 |
| 4 | condensed chromosome, centromeric region | GO:0000779 | 3.70E-08 |
| 4 | supramolecular complex | GO:0099080 | 3.70E-08 |
| 4 | DNA-binding transcription factor activity | GO:0003700 | 5.80E-07 |
| 4 | DASH complex | GO:0042729 | 1.90E-06 |
| 4 | nucleus | GO:0005634 | 2.80E-06 |
| 4 | regulation of gene expression | GO:0010468 | 7.90E-06 |
| 4 | regulation of nucleic acid-templated transcription | GO:1903506 | 1.10E-05 |
| 4 | cellular_component | GO:0006355 | 1.20E-05 |
| 4 | regulation of transcription, DNA-templated | GO:0006355 | 1.20E-05 |
| 4 | outer kinetochore | GO:0000940 | 1.40E-05 |
| 4 | mitotic spindle | GO:0072686 | 5.90E-05 |
| 4 | protein-containing complex | GO:0032991 | 6.00E-05 |
| 4 | histone deacetylase complex | GO:0000118 | 6.10E-04 |
| 4 | SHREC complex | GO:0070824 | 6.10E-04 |
| 4 | negative regulation of gene expression, epigenetic | GO:0045814 | 1.80E-03 |
| 4 | heterochromatin assembly | GO:0031507 | 1.80E-03 |
| 4 | heterochromatin organization | GO:0070828 | 1.80E-03 |
| 4 | spindle | GO:0005819 | 2.80E-03 |
| 4 | carboxylic acid binding | GO:0031406 | 4.10E-03 |
| 4 | L-ascorbic acid binding | GO:0031418 | 4.10E-03 |
| 4 | regulation of transcription by RNA polymerase II | GO:0006357 | 1.10E-02 |
| 4 | monosaccharide binding | GO:0048029 | 1.40E-02 |
| 4 | organic acid binding | GO:0043177 | 1.40E-02 |
| 4 | G protein-coupled receptor signaling pathway | GO:0007186 | 1.90E-02 |
| 4 | G protein-coupled receptor activity | GO:0004930 | 3.60E-02 |
| 3 | non-membrane-bounded organelle assembly | GO:0140694 | 5.90E-05 |
| 3 | organelle assembly | GO:0070925 | 1.30E-04 |
| 3 | nucleus | GO:0005634 | 2.00E-04 |
| 3 | Integrator complex | GO:0032039 | 5.70E-04 |
| 3 | kinetochore assembly | GO:0051382 | 9.80E-04 |
| 3 | kinetochore organization | GO:0051383 | 9.80E-04 |
| 3 | nuclear protein-containing complex | GO:0140513 | 3.40E-03 |
| 3 | protein binding | GO:0005515 | 5.30E-03 |
| 3 | centromere complex assembly | GO:0034508 | 5.60E-03 |
| 3 | snRNA processing | GO:0016180 | 1.30E-02 |
| 3 | snRNA metabolic process | GO:0016073 | 1.30E-02 |
| 3 | protein-DNA complex subunit organization | GO:0071824 | 4.20E-02 |
| 2 | protein binding | GO:0005515 | 1.00E-57 |
| 2 | binding | GO:0005488 | 3.50E-31 |
| 2 | nucleus | GO:0005634 | 1.10E-21 |
| 2 | protein-containing complex | GO:0032991 | 2.10E-18 |
| 2 | protein-containing complex assembly | GO:0065003 | 1.10E-14 |
| 2 | intracellular membrane-bounded organelle | GO:0043231 | 1.40E-14 |
| 2 | cellular component assembly | GO:0022607 | 4.10E-14 |
| 2 | protein-containing complex organization | GO:0043933 | 1.10E-13 |
| 2 | nuclear protein-containing complex | GO:0140513 | 1.30E-12 |
| 2 | protein homooligomerization | GO:0051260 | 7.50E-11 |
| 2 | protein complex oligomerization | GO:0051259 | 2.80E-10 |
| 2 | organelle membrane | GO:0031090 | 1.20E-09 |
| 2 | regulation of gene expression | GO:0010468 | 9.70E-09 |
| 2 | membrane-bounded organelle | GO:0043227 | 1.40E-08 |
| 2 | cellular_component | GO:0005575 | 3.70E-08 |
| 2 | integral component of organelle membrane | GO:0031301 | 4.00E-08 |
| 2 | cellular localization | GO:0051641 | 4.90E-08 |
| 2 | establishment of localization in cell | GO:0051649 | 1.30E-07 |
| 2 | regulation of nucleic acid-templated transcription | GO:1903506 | 1.30E-07 |
| 2 | regulation of transcription, DNA-templated | GO:0006355 | 1.60E-07 |
| 2 | intracellular transport | GO:0046907 | 1.70E-07 |
| 2 | intrinsic component of organelle membrane | GO:0031300 | 9.40E-07 |
| 2 | regulation of transcription by RNA polymerase II | GO:0006357 | 1.80E-06 |
| 2 | transcription coregulator activity | GO:0003712 | 1.60E-05 |
| 2 | microtubule cytoskeleton | GO:0015630 | 2.30E-05 |
| 2 | cellular component organization | GO:0016043 | 2.70E-05 |
| 2 | endomembrane system | GO:0012505 | 3.80E-05 |
| 2 | regulation of organelle organization | GO:0033043 | 5.20E-05 |
| 2 | regulation of cytoskeleton organization | GO:0051493 | 6.50E-05 |
| 2 | structural constituent of nuclear pore | GO:0017056 | 6.50E-05 |
| 2 | organelle organization | GO:0006996 | 8.60E-05 |
| 2 | DNA-binding transcription factor activity, RNA polymerase II-specific | GO:0000981 | 9.20E-05 |
| 2 | transcription regulator activity | GO:0140110 | 9.70E-05 |
| 2 | endoplasmic reticulum protein-containing complex | GO:0140534 | 1.80E-04 |
| 2 | regulation of cell cycle | GO:0051726 | 3.20E-04 |
| 2 | DNA-binding transcription factor activity | GO:0003700 | 3.40E-04 |
| 2 | biological process involved in interspecies interaction between organisms | GO:0044419 | 3.80E-04 |
| 2 | cytolysis in another organism | GO:0051715 | 3.80E-04 |
| 2 | killing of cells of another organism | GO:0031640 | 3.80E-04 |
| 2 | pore complex | GO:0046930 | 3.80E-04 |
| 2 | cell killing | GO:0001906 | 3.80E-04 |
| 2 | pore complex assembly | GO:0046931 | 3.80E-04 |
| 2 | protein targeting | GO:0006605 | 3.90E-04 |
| 2 | transcription, DNA-templated | GO:0006351 | 5.60E-04 |
| 2 | nuclear envelope | GO:0005635 | 6.10E-04 |
| 2 | envelope | GO:0031975 | 8.10E-04 |
| 2 | localization | GO:0051179 | 1.00E-03 |
| 2 | nucleic acid-templated transcription | GO:0097659 | 1.20E-03 |
| 2 | RNA polymerase complex | GO:0030880 | 1.30E-03 |
| 2 | organelle | GO:0043226 | 1.40E-03 |
| 2 | organelle envelope | GO:0031967 | 1.50E-03 |
| 2 | regulation of cellular component organization | GO:0051128 | 1.70E-03 |
| 2 | regulation of cell cycle process | GO:0010564 | 1.80E-03 |
| 2 | nuclear lumen | GO:0031981 | 2.10E-03 |
| 2 | positive regulation of protein kinase activity | GO:0045860 | 2.70E-03 |
| 2 | intrinsic component of nuclear inner membrane | GO:0031229 | 2.70E-03 |
| 2 | origin recognition complex | GO:0000808 | 2.70E-03 |
| 2 | integral component of nuclear inner membrane | GO:0005639 | 2.70E-03 |
| 2 | intracellular organelle | GO:0043229 | 2.90E-03 |
| 2 | intracellular protein transmembrane transport | GO:0065002 | 3.10E-03 |
| 2 | protein transmembrane transport | GO:0071806 | 3.10E-03 |
| 2 | peptidase complex | GO:1905368 | 3.70E-03 |
| 2 | cytoskeleton | GO:0005856 | 3.80E-03 |
| 2 | endoplasmic reticulum membrane | GO:0005789 | 4.90E-03 |
| 2 | protein transmembrane import into intracellular organelle | GO:0044743 | 5.90E-03 |
| 2 | nuclear pore | GO:0005643 | 5.90E-03 |
| 2 | protein localization | GO:0008104 | 6.80E-03 |
| 2 | regulation of intracellular signal transduction | GO:1902531 | 7.90E-03 |
| 2 | endoplasmic reticulum subcompartment | GO:0098827 | 1.10E-02 |
| 2 | mitochondrial protein-containing complex | GO:0098798 | 1.20E-02 |
| 2 | protein transport | GO:0015031 | 1.60E-02 |
| 2 | regulation of spindle organization | GO:0090224 | 1.70E-02 |
| 2 | activation of protein kinase activity | GO:0032147 | 1.70E-02 |
| 2 | regulation of mitotic spindle organization | GO:0060236 | 1.70E-02 |
| 2 | regulation of microtubule-based process | GO:0032886 | 1.70E-02 |
| 2 | regulation of microtubule cytoskeleton organization | GO:0070507 | 1.70E-02 |
| 2 | nuclear DNA-directed RNA polymerase complex | GO:0055029 | 1.80E-02 |
| 2 | nuclear outer membrane-endoplasmic reticulum membrane network | GO:0042175 | 1.80E-02 |
| 2 | DNA-directed RNA polymerase complex | GO:0000428 | 1.80E-02 |
| 2 | cell division | GO:0051301 | 1.90E-02 |
| 2 | membrane protein complex | GO:0098796 | 2.00E-02 |
| 2 | intracellular protein transport | GO:0006886 | 2.20E-02 |
| 2 | mitochondrion organization | GO:0007005 | 2.30E-02 |
| 2 | vesicle-mediated transport | GO:0016192 | 2.80E-02 |
| 2 | mediator complex | GO:0016592 | 2.90E-02 |
| 2 | cytolysis | GO:0019835 | 3.10E-02 |
| 2 | endoplasmic reticulum | GO:0005783 | 3.40E-02 |
| 2 | intracellular anatomical structure | GO:0005622 | 3.50E-02 |
| 1 | protein modification process | GO:0036211 | 5.0E-324 |
| 1 | macromolecule metabolic process | GO:0043170 | 5.0E-324 |
| 1 | metabolic process | GO:0008152 | 5.0E-324 |
| 1 | ATP binding | GO:0005524 | 5.0E-324 |
| 1 | organonitrogen compound metabolic process | GO:1901564 | 5.0E-324 |
| 1 | phosphotransferase activity, alcohol group as acceptor | GO:0016773 | 5.0E-324 |
| 1 | catalytic activity | GO:0003824 | 5.0E-324 |
| 1 | kinase activity | GO:0016301 | 5.0E-324 |
| 1 | primary metabolic process | GO:0044238 | 5.0E-324 |
| 1 | biological_process | GO:0008150 | 5.0E-324 |
| 1 | protein kinase activity | GO:0004672 | 5.0E-324 |
| 1 | adenyl nucleotide binding | GO:0030554 | 5.0E-324 |
| 1 | organic substance metabolic process | GO:0071704 | 5.0E-324 |
| 1 | catalytic activity, acting on a protein | GO:0140096 | 5.0E-324 |
| 1 | protein phosphorylation | GO:0006468 | 5.0E-324 |
| 1 | protein metabolic process | GO:0019538 | 5.0E-324 |
| 1 | purine ribonucleotide binding | GO:0032555 | 5.0E-324 |
| 1 | adenyl ribonucleotide binding | GO:0032559 | 5.0E-324 |
| 1 | phosphorylation | GO:0016310 | 5.0E-324 |
| 1 | purine ribonucleoside triphosphate binding | GO:0035639 | 5.0E-324 |
| 1 | macromolecule modification | GO:0043412 | 5.0E-324 |
| 1 | oxidoreductase activity | GO:0016491 | 2.20E-64 |
| 1 | ATP-dependent activity | GO:0140657 | 3.10E-64 |
| 1 | cellular process | GO:0009987 | 6.70E-57 |
| 1 | cellular metabolic process | GO:0044237 | 1.20E-53 |
| 1 | ion binding | GO:0043167 | 1.10E-50 |
| 1 | anion binding | GO:0043168 | 1.30E-49 |
| 1 | biosynthetic process | GO:0009058 | 3.50E-49 |
| 1 | hydrolase activity | GO:0016787 | 3.10E-40 |
| 1 | nucleoside phosphate binding | GO:1901265 | 2.40E-36 |
| 1 | molecular_function | GO:0003674 | 5.60E-36 |
| 1 | cellular biosynthetic process | GO:0044249 | 1.50E-33 |
| 1 | nucleoside-triphosphatase activity | GO:0017111 | 5.50E-33 |
| 1 | tetrapyrrole binding | GO:0046906 | 7.50E-27 |
| 1 | heme binding | GO:0020037 | 1.70E-26 |
| 1 | peptide biosynthetic process | GO:0043043 | 1.10E-25 |
| 1 | translation | GO:0006412 | 2.30E-25 |
| 1 | guanyl ribonucleotide binding | GO:0032561 | 2.50E-24 |
| 1 | GTP binding | GO:0005525 | 2.50E-24 |
| 1 | cellular macromolecule biosynthetic process | GO:0034645 | 6.30E-24 |
| 1 | hydrolase activity, acting on phosphorus-containing anhydrides | GO:0016818 | 7.70E-23 |
| 1 | transferase activity | GO:0016740 | 1.00E-22 |
| 1 | monooxygenase activity | GO:0004497 | 1.10E-22 |
| 1 | pyrophosphatase activity | GO:0016462 | 1.80E-22 |
| 1 | nitrogen compound metabolic process | GO:0006807 | 6.40E-22 |
| 1 | catalytic activity, acting on RNA | GO:0140098 | 2.50E-20 |
| 1 | iron ion binding | GO:0005506 | 7.60E-20 |
| 1 | ATP hydrolysis activity | GO:0016887 | 1.40E-19 |
| 1 | oxidoreductase activity | GO:0016705 | 1.70E-19 |
| 1 | carboxylic acid metabolic process | GO:0019752 | 2.90E-19 |
| 1 | small molecule metabolic process | GO:0044281 | 2.10E-18 |
| 1 | organic substance biosynthetic process | GO:1901576 | 2.30E-18 |
| 1 | cellular amino acid metabolic process | GO:0006520 | 6.40E-18 |
| 1 | oxoacid metabolic process | GO:0043436 | 1.00E-17 |
| 1 | transferase activity, transferring one-carbon groups | GO:0016741 | 2.80E-17 |
| 1 | gene expression | GO:0010467 | 3.00E-17 |
| 1 | macromolecule biosynthetic process | GO:0009059 | 5.20E-17 |
| 1 | catalytic activity, acting on a nucleic acid | GO:0140640 | 6.20E-17 |
| 1 | protein serine/threonine kinase activity | GO:0004674 | 2.20E-16 |
| 1 | methyltransferase activity | GO:0008168 | 8.80E-16 |
| 1 | flavin adenine dinucleotide binding | GO:0050660 | 1.30E-15 |
| 1 | organonitrogen compound biosynthetic process | GO:1901566 | 2.30E-15 |
| 1 | carbohydrate metabolic process | GO:0005975 | 7.60E-15 |
| 1 | active transmembrane transporter activity | GO:0022804 | 1.80E-14 |
| 1 | small molecule binding | GO:0036094 | 1.80E-14 |
| 1 | ribosome | GO:0005840 | 2.50E-14 |
| 1 | nucleotide binding | GO:0000166 | 6.50E-14 |
| 1 | sulfur compound metabolic process | GO:0006790 | 5.60E-13 |
| 1 | GTPase activity | GO:0003924 | 4.40E-12 |
| 1 | structural molecule activity | GO:0005198 | 7.40E-12 |
| 1 | protein tyrosine kinase activity | GO:0004713 | 1.70E-11 |
| 1 | catalytic activity, acting on a tRNA | GO:0140101 | 2.70E-11 |
| 1 | structural constituent of ribosome | GO:0003735 | 3.50E-11 |
| 1 | transmembrane transport | GO:0055085 | 6.10E-11 |
| 1 | glycosyltransferase activity | GO:0016757 | 7.60E-11 |
| 1 | alpha-amino acid metabolic process | GO:1901605 | 1.20E-10 |
| 1 | helicase activity | GO:0004386 | 2.40E-10 |
| 1 | ATP-dependent protein folding chaperone | GO:0140662 | 3.50E-10 |
| 1 | inorganic cation transmembrane transporter activity | GO:0022890 | 4.20E-10 |
| 1 | primary active transmembrane transporter activity | GO:0015399 | 5.30E-10 |
| 1 | phosphorus metabolic process | GO:0006793 | 1.50E-09 |
| 1 | organic acid metabolic process | GO:0006082 | 1.70E-09 |
| 1 | FAD binding | GO:0071949 | 4.10E-09 |
| 1 | ATPase-coupled transmembrane transporter activity | GO:0042626 | 4.50E-09 |
| 1 | protein folding chaperone | GO:0044183 | 4.50E-09 |
| 1 | lipid biosynthetic process | GO:0008610 | 6.80E-09 |
| 1 | lipid metabolic process | GO:0006629 | 9.80E-09 |
| 1 | oxidoreductase activity, acting on CH-OH group of donors | GO:0016614 | 1.50E-08 |
| 1 | organophosphate metabolic process | GO:0019637 | 1.60E-08 |
| 1 | peptidase activity | GO:0008233 | 1.80E-08 |
| 1 | hydrolase activity, acting on carbon-nitrogen (but not peptide) bonds | GO:0016810 | 2.40E-08 |
| 1 | cell surface receptor signaling pathway | GO:0007166 | 3.90E-08 |
| 1 | cellular modified amino acid metabolic process | GO:0006575 | 5.70E-08 |
| 1 | UDP-glycosyltransferase activity | GO:0008194 | 5.80E-08 |
| 1 | cation transmembrane transporter activity | GO:0008324 | 1.00E-07 |
| 1 | cellular nitrogen compound metabolic process | GO:0034641 | 2.80E-07 |
| 1 | tRNA aminoacylation | GO:0043039 | 3.40E-07 |
| 1 | acyltransferase activity | GO:0016746 | 3.70E-07 |
| 1 | tRNA aminoacylation for protein translation | GO:0006418 | 5.20E-07 |
| 1 | ligase activity, forming carbon-oxygen bonds | GO:0016875 | 5.20E-07 |
| 1 | aminoacyl-tRNA ligase activity | GO:0004812 | 5.20E-07 |
| 1 | magnesium ion binding | GO:0000287 | 8.10E-07 |
| 1 | glutathione metabolic process | GO:0006749 | 8.10E-07 |
| 1 | vitamin B6 binding | GO:0070279 | 8.10E-07 |
| 1 | pyridoxal phosphate binding | GO:0030170 | 8.10E-07 |
| 1 | active ion transmembrane transporter activity | GO:0022853 | 1.10E-06 |
| 1 | signal transduction | GO:0007165 | 1.20E-06 |
| 1 | cellular amino acid biosynthetic process | GO:0008652 | 1.90E-06 |
| 1 | response to stimulus | GO:0050896 | 2.30E-06 |
| 1 | cellular amide metabolic process | GO:0043603 | 2.60E-06 |
| 1 | isomerase activity | GO:0016853 | 3.00E-06 |
| 1 | proteolysis | GO:0006508 | 3.60E-06 |
| 1 | metal ion transmembrane transporter activity | GO:0046873 | 4.70E-06 |
| 1 | organic cyclic compound metabolic process | GO:1901360 | 7.60E-06 |
| 1 | organophosphate biosynthetic process | GO:0090407 | 8.90E-06 |
| 1 | ligase activity | GO:0016874 | 8.90E-06 |
| 1 | transporter activity | GO:0005215 | 8.90E-06 |
| 1 | ATP-dependent activity, acting on RNA | GO:0008186 | 1.10E-05 |
| 1 | RNA helicase activity | GO:0003724 | 1.10E-05 |
| 1 | carboxylic acid biosynthetic process | GO:0046394 | 1.10E-05 |
| 1 | phosphate-containing compound metabolic process | GO:0006796 | 1.20E-05 |
| 1 | oxidoreductase activity, acting on the CH-CH group of donors | GO:0016627 | 2.00E-05 |
| 1 | monocarboxylic acid metabolic process | GO:0032787 | 2.70E-05 |
| 1 | iron-sulfur cluster binding | GO:0051536 | 2.70E-05 |
| 1 | oxidoreductase activity, acting on the CH-OH group of donors | GO:0016616 | 2.70E-05 |
| 1 | metal cluster binding | GO:0051540 | 2.70E-05 |
| 1 | metallopeptidase activity | GO:0008237 | 3.70E-05 |
| 1 | RNA modification | GO:0009451 | 3.90E-05 |
| 1 | ABC-type transporter activity | GO:0140359 | 4.20E-05 |
| 1 | cellular anatomical entity | GO:0110165 | 4.80E-05 |
| 1 | macromolecule methylation | GO:0043414 | 7.10E-05 |
| 1 | cytoskeletal motor activity | GO:0003774 | 1.00E-04 |
| 1 | alpha-amino acid biosynthetic process | GO:1901607 | 1.00E-04 |
| 1 | metal ion transport | GO:0030001 | 1.20E-04 |
| 1 | cell communication | GO:0007154 | 1.20E-04 |
| 1 | signaling | GO:0023052 | 1.20E-04 |
| 1 | ATP-dependent activity, acting on DNA | GO:0008094 | 1.30E-04 |
| 1 | small molecule biosynthetic process | GO:0044283 | 1.60E-04 |
| 1 | carbon-oxygen lyase activity | GO:0016835 | 1.60E-04 |
| 1 | NAD binding | GO:0051287 | 2.40E-04 |
| 1 | nucleoside phosphate metabolic process | GO:0006753 | 2.40E-04 |
| 1 | hydro-lyase activity | GO:0016836 | 2.40E-04 |
| 1 | nucleotide metabolic process | GO:0009117 | 2.40E-04 |
| 1 | organic acid biosynthetic process | GO:0016053 | 2.40E-04 |
| 1 | phosphoric ester hydrolase activity | GO:0042578 | 2.50E-04 |
| 1 | cellular response to stimulus | GO:0051716 | 2.60E-04 |
| 1 | cation transport | GO:0006812 | 2.70E-04 |
| 1 | nucleobase-containing small molecule metabolic process | GO:0055086 | 3.80E-04 |
| 1 | hydrolase activity, acting on glycosyl bonds | GO:0016798 | 3.80E-04 |
| 1 | carbohydrate derivative metabolic process | GO:1901135 | 4.50E-04 |
| 1 | hexosyltransferase activity | GO:0016758 | 4.60E-04 |
| 1 | cellular lipid metabolic process | GO:0044255 | 4.80E-04 |
| 1 | peptide metabolic process | GO:0006518 | 4.90E-04 |
| 1 | catabolic process | GO:0009056 | 5.50E-04 |
| 1 | ATPase-coupled cation transmembrane transporter activity | GO:0019829 | 5.60E-04 |
| 1 | hydrolase activity, acting on carbon-nitrogen in linear amides | GO:0016811 | 5.80E-04 |
| 1 | carbon-carbon lyase activity | GO:0016830 | 5.80E-04 |
| 1 | lyase activity | GO:0016829 | 5.80E-04 |
| 1 | phosphoprotein phosphatase activity | GO:0004721 | 8.20E-04 |
| 1 | S-adenosylmethionine-dependent methyltransferase activity | GO:0008757 | 9.10E-04 |
| 1 | carboxylic ester hydrolase activity | GO:0052689 | 9.10E-04 |
| 1 | transmembrane transporter activity | GO:0022857 | 1.20E-03 |
| 1 | ribosomal subunit | GO:0044391 | 1.30E-03 |
| 1 | anion transmembrane transporter activity | GO:0008509 | 1.40E-03 |
| 1 | exopeptidase activity | GO:0008238 | 1.40E-03 |
| 1 | microtubule motor activity | GO:0003777 | 1.40E-03 |
| 1 | serine-type endopeptidase activity | GO:0004252 | 1.80E-03 |
| 1 | phosphatase activity | GO:0016791 | 2.30E-03 |
| 1 | organic substance catabolic process | GO:1901575 | 2.50E-03 |
| 1 | transferase activity, transferring phosphorus-containing groups | GO:0016772 | 2.60E-03 |
| 1 | structural constituent of chromatin | GO:0030527 | 2.90E-03 |
| 1 | cellular aromatic compound metabolic process | GO:0006725 | 3.00E-03 |
| 1 | RNA methylation | GO:0001510 | 3.40E-03 |
| 1 | oxidoreductase activity, acting on the aldehyde or oxo group of donors | GO:0016903 | 3.40E-03 |
| 1 | protein folding | GO:0006457 | 3.40E-03 |
| 1 | proton transmembrane transporter activity | GO:0015078 | 4.20E-03 |
| 1 | organic cyclic compound biosynthetic process | GO:1901362 | 4.80E-03 |
| 1 | hydrolase activity, hydrolyzing O-glycosyl compounds | GO:0004553 | 5.20E-03 |
| 1 | nucleotide biosynthetic process | GO:0009165 | 5.20E-03 |
| 1 | aromatic amino acid family metabolic process | GO:0009072 | 5.20E-03 |
| 1 | phospholipid metabolic process | GO:0006644 | 5.70E-03 |
| 1 | acetyltransferase activity | GO:0016407 | 6.60E-03 |
| 1 | intracellular anatomical structure | GO:0005622 | 7.50E-03 |
| 1 | intramolecular transferase activity | GO:0016866 | 8.10E-03 |
| 1 | inorganic anion transmembrane transporter activity | GO:0015103 | 8.10E-03 |
| 1 | cation transmembrane transport | GO:0098655 | 8.60E-03 |
| 1 | inorganic ion transmembrane transport | GO:0098660 | 8.60E-03 |
| 1 | translation factor activity, RNA binding | GO:0008135 | 9.20E-03 |
| 1 | nucleobase-containing compound metabolic process | GO:0006139 | 9.40E-03 |
| 1 | carbohydrate derivative biosynthetic process | GO:1901137 | 9.60E-03 |
| 1 | heterocycle metabolic process | GO:0046483 | 9.80E-03 |
| 1 | microtubule-based movement | GO:0007018 | 9.90E-03 |
| 1 | cellular catabolic process | GO:0044248 | 1.00E-02 |
| 1 | serine hydrolase activity | GO:0017171 | 1.10E-02 |
| 1 | serine-type peptidase activity | GO:0008236 | 1.10E-02 |
| 1 | inorganic cation transmembrane transport | GO:0098662 | 1.20E-02 |
| 1 | fatty acid metabolic process | GO:0006631 | 1.30E-02 |
| 1 | N-acyltransferase activity | GO:0016410 | 1.50E-02 |
| 1 | serine family amino acid metabolic process | GO:0009069 | 1.90E-02 |
| 1 | sulfur amino acid metabolic process | GO:0000096 | 1.90E-02 |
| 1 | methylation | GO:0032259 | 2.20E-02 |
| 1 | anion transport | GO:0006820 | 2.20E-02 |
| 1 | coated membrane | GO:0048475 | 2.20E-02 |
| 1 | membrane coat | GO:0030117 | 2.20E-02 |
| 1 | protein dephosphorylation | GO:0006470 | 2.60E-02 |
| 1 | deubiquitinase activity | GO:0101005 | 2.90E-02 |
| 1 | nucleoside phosphate biosynthetic process | GO:1901293 | 3.00E-02 |
| 1 | phospholipid biosynthetic process | GO:0008654 | 3.30E-02 |
| 1 | purine-containing compound metabolic process | GO:0072521 | 4.70E-02 |
| 1 | oxidoreductase activity, acting on the CH-CH group of donors | GO:0016628 | 4.70E-02 |
| 1 | carboxy-lyase activity | GO:0016831 | 4.70E-02 |
| 1 | glutamine family amino acid metabolic process | GO:0009064 | 4.70E-02 |
| 1 | RNA methyltransferase activity | GO:0008173 | 4.70E-02 |
| 1 | tricarboxylic acid cycle | GO:0006099 | 4.70E-02 |
| 1 | carbohydrate derivative binding | GO:0097367 | 5.00E-02 |
| 1 | N-acetyltransferase activity | GO:0008080 | 5.00E-02 |

**Table S2.** Top 15 InterPro domains found at each phylorank

|  | **All genes** | **High-confidence genes** | | | | |
| --- | --- | --- | --- | --- | --- | --- |
| **Phylo rank** | **InterPro domain** | **Description** | **Count** | **InterPro domain** | **Description** | **Count** |
| 1 | ipr011009 | Protein kinase-like | 2189 | — | — | — |
| 1 | ipr000719 | Protein kinase | 1865 | — | — | — |
| 1 | ipr001245 | Serine-threonine/tyrosine-protein kinase | 1551 | — | — | — |
| 1 | ipr011990 | Tetratricopeptide-like helical domain | 716 | — | — | — |
| 1 | ipr027417 | P-loop containing nucleoside triphosphate hydrolase | 642 | — | — | — |
| 1 | ipr006597 | Sel1-like repeat | 607 | — | — | — |
| 1 | ipr017441 | Protein kinase, ATP binding site | 508 | — | — | — |
| 1 | ipr011333 | SKP1/BTB/POZ | 251 | — | — | — |
| 1 | ipr000210 | BTB/POZ | 239 | — | — | — |
| 1 | ipr006571 | TLDc domain | 226 | — | — | — |
| 1 | ipr029063 | S-adenosyl-L-methionine-dependent methyltransferase | 183 | — | — | — |
| 1 | ipr015943 | WD40/YVTN repeat-like-containing domain superfamily | 170 | — | — | — |
| 1 | ipr036291 | NAD(P)-binding domain superfamily | 164 | — | — | — |
| 1 | ipr043129 | ATPase, nucleotide binding domain | 162 | — | — | — |
| 1 | ipr001128 | Cytochrome P450 | 151 | — | — | — |
| 2 | ipr011333 | SKP1/BTB/POZ | 554 | — | — | — |
| 2 | ipr000210 | BTB/POZ | 525 | — | — | — |
| 2 | ipr006571 | TLDc domain | 378 | — | — | — |
| 2 | ipr011705 | BTB/Kelch-associated | 267 | — | — | — |
| 2 | ipr013320 | Concanavalin A-like lectin/glucanase | 111 | — | — | — |
| 2 | ipr043136 | B30.2/SPRY domain superfamily | 107 | — | — | — |
| 2 | ipr027417 | P-loop containing nucleoside triphosphate hydrolase | 91 | — | — | — |
| 2 | ipr045379 | Crinkler (CRN) family protein | 88 | — | — | — |
| 2 | ipr001870 | B30.2/SPRY domain. | 86 | — | — | — |
| 2 | ipr003877 | SPRY domain | 80 | — | — | — |
| 2 | ipr011990 | Tetratricopeptide-like helical domain | 46 | — | — | — |
| 2 | ipr043502 | DNA/RNA polymerase superfamily | 46 | — | — | — |
| 2 | ipr011009 | Protein kinase-like | 45 | — | — | — |
| 2 | ipr036322 | WD40-repeat-containing domain superfamily | 44 | — | — | — |
| 2 | ipr015915 | Kelch-type beta propeller | 43 | — | — | — |
| 3 | ipr011333 | SKP1/BTB/POZ | 67 | — | — | — |
| 3 | ipr000210 | BTB/POZ | 59 | — | — | — |
| 3 | ipr009057 | Homeobox-like domain superfamily | 18 | — | — | — |
| 3 | ipr011705 | BTB/Kelch-associated | 14 | — | — | — |
| 3 | ipr006571 | TLDc domain | 13 | — | — | — |
| 3 | ipr036388 | Winged helix-like DNA-binding domain superfamily | 11 | — | — | — |
| 3 | ipr040410 | UPF0658 Golgi apparatus membrane protein | 9 | — | — | — |
| 3 | ipr011009 | Protein kinase-like | 7 | — | — | — |
| 3 | ipr011990 | Tetratricopeptide-like helical domain | 7 | — | — | — |
| 3 | ipr014752 | Arrestin-like_C | 7 | — | — | — |
| 3 | ipr014756 | Immunoglobulin E-set | 7 | — | — | — |
| 3 | ipr032675 | Leucine-rich | 7 | — | — | — |
| 3 | ipr036910 | High mobility group box domain superfamily | 7 | — | — | — |
| 3 | ipr000719 | Protein kinase | 6 | — | — | — |
| 3 | ipr006597 | Sel1-like repeat | 6 | — | — | — |
| 4 | ipr036047 | F-box-like domain superfamily | 41 | ipr003108 | GAR domain | 3 |
| 4 | ipr001810 | F-box domain | 37 | ipr018159 | Spectrin/alpha-actinin | 3 |
| 4 | ipr032675 | Leucine-rich | 24 | ipr036534 | GAR domain superfamily | 3 |
| 4 | ipr009057 | Homeobox-like domain superfamily | 23 | ipr001810 | F-box domain | 1 |
| 4 | ipr045379 | Crinkler (CRN) family protein | 23 | ipr001878 | Zinc finger, CCHC-type | 1 |
| 4 | ipr036910 | High mobility group box domain superfamily | 22 | ipr001895 | Ras guanine-nucleotide exchange factors catalytic domain | 1 |
| 4 | ipr036300 | Mir domain superfamily | 17 | ipr001965 | Zinc finger, PHD-type | 1 |
| 4 | ipr016093 | MIR motif | 16 | ipr002110 | Ankyrin repeat | 1 |
| 4 | ipr009071 | High mobility group box domain | 15 | ipr003163 | Transcription regulator HTH, APSES-type DNA-binding domain | 1 |
| 4 | ipr011333 | SKP1/BTB/POZ | 14 | ipr003657 | WRKY domain | 1 |
| 4 | ipr004827 | Basic-leucine zipper domain | 11 | ipr008952 | Tetraspanin, EC2 domain superfamily | 1 |
| 4 | ipr011990 | Tetratricopeptide-like helical domain | 11 | ipr009818 | Ataxin-2, C-terminal | 1 |
| 4 | ipr017452 | GPCR, rhodopsin-like, 7TM | 11 | ipr011011 | Zinc finger, FYVE/PHD-type | 1 |
| 4 | ipr046347 | Basic-leucine zipper domain superfamily | 10 | ipr013083 | Zinc finger, RING/FYVE/PHD-type | 1 |
| 4 | ipr000210 | BTB/POZ | 9 | ipr013726 | Protein of unknown function DUF1748, fungi | 1 |
| 5 | ipr035979 | RNA-binding domain superfamily | 34 | ipr032675 | Leucine-rich | 6 |
| 5 | ipr024862 | Transient receptor potential cation channel subfamily V | 32 | ipr036047 | F-box-like domain superfamily | 4 |
| 5 | ipr012677 | Nucleotide-binding alpha-beta plait domain superfamily | 26 | ipr001810 | F-box domain | 3 |
| 5 | ipr000504 | RNA recognition motif domain | 19 | ipr013320 | Concanavalin A-like lectin/glucanase | 3 |
| 5 | ipr032675 | Leucine-rich | 15 | ipr011425 | Mediator of RNA polymerase II transcription subunit 9 | 2 |
| 5 | ipr045379 | Crinkler (CRN) family protein | 15 | ipr000210 | BTB/POZ | 1 |
| 5 | ipr001878 | Zinc finger, CCHC-type | 14 | ipr001611 | Leucine-rich repeats | 1 |
| 5 | ipr016093 | MIR motif | 14 | ipr001995 | Peptidase A2A, retrovirus, catalytic | 1 |
| 5 | ipr036300 | Mir domain superfamily | 14 | ipr008538 | Putative restriction endonuclease | 1 |
| 5 | ipr005821 | Ion transport domain | 11 | ipr008626 | Mediator complex, subunit Med15, fungi | 1 |
| 5 | ipr011333 | SKP1/BTB/POZ | 9 | ipr008936 | Rho GTPase activation protein | 1 |
| 5 | ipr027417 | P-loop containing nucleoside triphosphate hydrolase | 8 | ipr011021 | Arrestin-like, N-terminal | 1 |
| 5 | ipr011009 | Protein kinase-like | 7 | ipr011333 | SKP1/BTB/POZ | 1 |
| 5 | ipr013320 | Concanavalin A-like lectin/glucanase | 7 | ipr011990 | Tetratricopeptide-like helical domain | 1 |
| 5 | ipr036047 | F-box-like domain superfamily | 7 | ipr012296 | Nuclease, putative, TT1808 | 1 |
| 6 | ipr032675 | Leucine-rich | 652 | ipr032675 | Leucine-rich | 21 |
| 6 | ipr036047 | F-box-like domain superfamily | 144 | ipr001810 | F-box domain | 16 |
| 6 | ipr011990 | Tetratricopeptide-like helical domain | 141 | ipr036047 | F-box-like domain superfamily | 14 |
| 6 | ipr001810 | F-box domain | 133 | ipr021109 | Aspartic peptidase | 6 |
| 6 | ipr036910 | High mobility group box domain superfamily | 128 | ipr036910 | High mobility group box domain superfamily | 4 |
| 6 | ipr006597 | Sel1-like repeat | 95 | ipr003450 | Replication origin-binding protein | 3 |
| 6 | ipr009071 | High mobility group box domain | 75 | ipr009071 | High mobility group box domain | 3 |
| 6 | ipr001878 | Zinc finger, CCHC-type | 49 | ipr000719 | Protein kinase | 2 |
| 6 | ipr036300 | Mir domain superfamily | 49 | ipr009057 | Homeobox-like domain superfamily | 2 |
| 6 | ipr011009 | Protein kinase-like | 47 | ipr011009 | Protein kinase-like | 2 |
| 6 | ipr013083 | Zinc finger, RING/FYVE/PHD-type | 46 | ipr011990 | Tetratricopeptide-like helical domain | 2 |
| 6 | ipr016093 | MIR motif | 46 | ipr045379 | Crinkler (CRN) family protein | 2 |
| 6 | ipr013761 | Sterile alpha motif/pointed domain superfamily | 40 | ipr000812 | Transcription factor TFIIB | 1 |
| 6 | ipr036875 | Zinc finger, CCHC-type superfamily | 40 | ipr001005 | SANT/Myb domain | 1 |
| 6 | ipr011333 | SKP1/BTB/POZ domain superfamily | 37 | ipr001138 | Zn(2)-C6 fungal-type DNA-binding domain | 1 |
| 7 | ipr011990 | Tetratricopeptide-like helical domain | 196 | ipr000504 | RNA recognition motif domain | 2 |
| 7 | ipr006597 | Sel1-like repeat | 189 | ipr006597 | Sel1-like repeat | 2 |
| 7 | ipr036910 | High mobility group box domain superfamily | 50 | ipr011990 | Tetratricopeptide-like helical domain | 2 |
| 7 | ipr009057 | Homeobox-like domain superfamily | 32 | ipr012677 | Nucleotide-binding alpha-beta plait domain superfamily | 2 |
| 7 | ipr009071 | High mobility group box domain | 21 | ipr032675 | Leucine-rich | 2 |
| 7 | ipr004088 | K Homology domain, type 1 | 20 | ipr035979 | RNA-binding domain superfamily | 2 |
| 7 | ipr036612 | K Homology domain, type 1 superfamily | 20 | ipr001214 | SET domain | 1 |
| 7 | ipr045379 | Crinkler (CRN) family protein | 19 | ipr001810 | F-box domain | 1 |
| 7 | ipr001005 | SANT/Myb domain | 17 | ipr004827 | Basic-leucine zipper domain | 1 |
| 7 | ipr021109 | Aspartic peptidase | 12 | ipr007268 | Rad9/Ddc1 | 1 |
| 7 | ipr011009 | Protein kinase-like | 11 | ipr019734 | Tetratricopeptide repeat | 1 |
| 7 | ipr017930 | Myb domain | 11 | ipr021109 | Aspartic peptidase | 1 |
| 7 | ipr035979 | RNA-binding domain superfamily | 10 | ipr036047 | F-box-like domain superfamily | 1 |
| 7 | ipr032675 | Leucine-rich | 9 | ipr036063 | Smr domain superfamily | 1 |
| 7 | ipr002068 | Alpha crystallin/Hsp20 domain | 8 | ipr036389 | Ribonuclease III, endonuclease domain superfamily | 1 |
| 8 | ipr001810 | F-box domain | 87 | ipr006597 | Sel1-like repeat | 4 |
| 8 | ipr036047 | F-box-like domain superfamily | 57 | ipr011990 | Tetratricopeptide-like helical domain | 4 |
| 8 | ipr006597 | Sel1-like repeat | 33 | ipr000219 | Dbl homology (DH) domain | 1 |
| 8 | ipr011990 | Tetratricopeptide-like helical domain | 33 | ipr001138 | Zn(2)-C6 fungal-type DNA-binding domain | 1 |
| 8 | ipr036910 | High mobility group box domain superfamily | 12 | ipr004827 | Basic-leucine zipper domain | 1 |
| 8 | ipr036875 | Zinc finger, CCHC-type superfamily | 9 | ipr008265 | Lipase, GDSL, active site | 1 |
| 8 | ipr001878 | Zinc finger, CCHC-type | 8 | ipr009057 | Homeobox-like domain superfamily | 1 |
| 8 | ipr032675 | Leucine-rich | 8 | ipr009071 | High mobility group box domain | 1 |
| 8 | ipr045379 | Crinkler (CRN) family protein | 6 | ipr019734 | Tetratricopeptide repeat | 1 |
| 8 | ipr009071 | High mobility group box domain | 4 | ipr036388 | Winged helix-like DNA-binding domain superfamily | 1 |
| 8 | ipr009057 | Homeobox-like domain superfamily | 3 | ipr036864 | Zn(2)-C6 fungal-type DNA-binding domain superfamily | 1 |
| 8 | ipr001005 | SANT/Myb domain | 2 | ipr036910 | High mobility group box domain superfamily | 1 |
| 8 | ipr004088 | K Homology domain, type 1 | 2 | ipr046347 | Basic-leucine zipper domain superfamily | 1 |
| 8 | ipr004827 | Basic-leucine zipper domain | 2 | — | — | — |
| 8 | ipr004968 | DNA primase/nucleoside triphosphatase, C-terminal | 2 | — | — | — |
| 9 | ipr011990 | Tetratricopeptide-like helical domain | 15 | — | — | — |
| 9 | ipr006597 | Sel1-like repeat | 13 | — | — | — |
| 9 | ipr032675 | Leucine-rich | 3 | — | — | — |
| 9 | ipr004827 | Basic-leucine zipper domain | 2 | — | — | — |
| 9 | ipr013761 | Sterile alpha motif/pointed domain superfamily | 2 | — | — | — |
| 9 | ipr016133 | Insect cysteine-rich antifreeze protein | 2 | — | — | — |
| 9 | ipr027417 | P-loop containing nucleoside triphosphate hydrolase | 2 | — | — | — |
| 9 | ipr035992 | Ricin B-like lectins | 2 | — | — | — |
| 9 | ipr046347 | Basic-leucine zipper domain superfamily | 2 | — | — | — |
| 9 | ipr000651 | Ras-like guanine nucleotide exchange factor, N-terminal | 1 | — | — | — |
| 9 | ipr001289 | Nuclear transcription factor Y subunit A | 1 | — | — | — |
| 9 | ipr001611 | Leucine-rich repeats | 1 | — | — | — |
| 9 | ipr002885 | Pentatricopeptide repeat | 1 | — | — | — |
| 9 | ipr003323 | OTU domain | 1 | — | — | — |
| 9 | ipr004968 | DNA primase/nucleoside triphosphatase, C-terminal | 1 | — | — | — |

**Table S3.** High-confidence Glomeromycetes-restricted genes

| **Gene ID** | **PFAM** | **InterPro** | **GO Terms** | **Secreted** | **Anti SMASH** |
| --- | --- | --- | --- | --- | --- |
| g14832 | NA | IPR000719 Protein kinase domain;IPR011009 Protein kinase-like domain superfamily | GO:0004672 - protein kinase activity ;GO:0005524 - ATP binding ;GO_process: GO:0006468 - protein phosphorylation | NA | NA |
| g5560 | NA | IPR000719 Protein kinase domain;IPR011009 Protein kinase-like domain superfamily | GO:0004672 - protein kinase activity ;GO:0005524 - ATP binding ;GO_process: GO:0006468 - protein phosphorylation | NA | NA |
| g8058 | NA | IPR000812 Transcription factor TFIIB | GO_process: GO:0070897 - transcription preinitiation complex assembly ;GO_process: GO:0006352 - DNA-templated transcription, initiation | NA | NA |
| g25709 | NA | IPR001005 SANT/Myb domain;IPR009057 Homeobox-like domain superfamily | NA | NA | NA |
| g25112 | PF00172 | IPR001138 Zn(2)-C6 fungal-type DNA-binding domain;IPR036864 Zn(2)-C6 fungal-type DNA-binding domain superfamily | GO:0008270 - zinc ion binding ;GO:0000981 - DNA-binding transcription factor activity, RNA polymerase II-specific ;GO_process: GO:0006355 - regulation of transcription, DNA-templated | NA | NA |
| g18154 | PF12937 | IPR001810 F-box domain;IPR032675 Leucine-rich repeat domain superfamily | GO:0005515 - protein binding | NA | NA |
| g19958 | PF12937 | IPR001810 F-box domain;IPR032675 Leucine-rich repeat domain superfamily | GO:0005515 - protein binding | NA | Cluster_3 |
| g31114 | PF12937 | IPR001810 F-box domain;IPR032675 Leucine-rich repeat domain superfamily | GO:0005515 - protein binding | SignalP(1-21,cutsite=TAA-DT,prob=0.5618) | NA |
| g4457 | PF12937 | IPR001810 F-box domain;IPR032675 Leucine-rich repeat domain superfamily | GO:0005515 - protein binding | NA | NA |
| g6802 | PF12937 | IPR001810 F-box domain;IPR032675 Leucine-rich repeat domain superfamily | GO:0005515 - protein binding | NA | NA |
| g8165 | PF12937 | IPR001810 F-box domain;IPR032675 Leucine-rich repeat domain superfamily | GO:0005515 - protein binding | NA | NA |
| g12531 | PF12937 | IPR001810 F-box domain;IPR032675 Leucine-rich repeat domain superfamily;IPR036047 F-box-like domain superfamily | GO:0005515 - protein binding | NA | NA |
| g16 | PF12937 | IPR001810 F-box domain;IPR032675 Leucine-rich repeat domain superfamily;IPR036047 F-box-like domain superfamily | GO:0005515 - protein binding | NA | NA |
| g25032 | PF12937 | IPR001810 F-box domain;IPR032675 Leucine-rich repeat domain superfamily;IPR036047 F-box-like domain superfamily | GO:0005515 - protein binding | NA | NA |
| g28343 | PF12937 | IPR001810 F-box domain;IPR032675 Leucine-rich repeat domain superfamily;IPR036047 F-box-like domain superfamily | GO:0005515 - protein binding | NA | NA |
| g15302 | NA | IPR001810 F-box domain;IPR036047 F-box-like domain superfamily | GO:0005515 - protein binding | NA | NA |
| g18383 | NA | IPR001810 F-box domain;IPR036047 F-box-like domain superfamily | GO:0005515 - protein binding | NA | NA |
| g19511 | PF00646 | IPR001810 F-box domain;IPR036047 F-box-like domain superfamily | GO:0005515 - protein binding | NA | NA |
| g31167 | NA | IPR001810 F-box domain;IPR036047 F-box-like domain superfamily | GO:0005515 - protein binding | NA | NA |
| g5731 | PF12937 | IPR001810 F-box domain;IPR036047 F-box-like domain superfamily | GO:0005515 - protein binding | NA | NA |
| g25877 | PF00646 | IPR001810 F-box domain;IPR036047 F-box-like domain superfamily;IPR036322 WD40-repeat-containing domain superfamily | GO:0005515 - protein binding | NA | NA |
| g1488 | NA | IPR001878 Zinc finger, CCHC-type;IPR021109 Aspartic peptidase domain superfamily;IPR036875 Zinc finger, CCHC-type superfamily | GO:0008270 - zinc ion binding ;GO:0003676 - nucleic acid binding | NA | NA |
| g31123 | PF01713 | IPR002625 Smr domain;IPR036063 Smr domain superfamily | NA | NA | NA |
| g4815 | NA | IPR003163 Transcription regulator HTH, APSES-type DNA-binding domain;IPR036887 HTH APSES-type DNA-binding domain superfamily | GO:0003677 - DNA binding | NA | NA |
| g15381 | NA | IPR003450 Replication origin-binding protein | GO:0003688 - DNA replication origin binding ;GO:0005524 - ATP binding ;GO_process: GO:0006260 - DNA replication | NA | NA |
| g20925 | NA | IPR003450 Replication origin-binding protein | GO:0003688 - DNA replication origin binding ;GO:0005524 - ATP binding ;GO_process: GO:0006260 - DNA replication | NA | NA |
| g2621 | NA | IPR003450 Replication origin-binding protein | GO:0003688 - DNA replication origin binding ;GO:0005524 - ATP binding ;GO_process: GO:0006260 - DNA replication | NA | NA |
| g3676 | PF08238 | IPR006597 Sel1-like repeat;IPR011990 Tetratricopeptide-like helical domain superfamily | GO:0005515 - protein binding | NA | NA |
| g6617 | PF05347 | IPR008011 Complex 1 LYR protein domain | NA | NA | NA |
| g21791 | NA | IPR009057 Homeobox-like domain superfamily | NA | NA | NA |
| g11504 | NA | IPR009071 High mobility group box domain;IPR036910 High mobility group box domain superfamily | NA | NA | NA |
| g29493 | PF00505; PF09011 | IPR009071 High mobility group box domain;IPR036910 High mobility group box domain superfamily | NA | NA | NA |
| g31092 | PF00505; PF09011 | IPR009071 High mobility group box domain;IPR036910 High mobility group box domain superfamily | NA | NA | NA |
| g11934 | NA | IPR011990 Tetratricopeptide-like helical domain superfamily | GO:0005515 - protein binding | NA | NA |
| g20642 | NA | IPR013783 Immunoglobulin-like fold | NA | NA | NA |
| g2640 | PF09463 | IPR018571 Membrane anchor Opy2, N-terminal | NA | SignalP(1-30,cutsite=VTA-QE,prob=0.9581) | NA |
| g12978 | NA | IPR021109 Aspartic peptidase domain superfamily | NA | NA | NA |
| g24870 | NA | IPR021109 Aspartic peptidase domain superfamily | NA | NA | NA |
| g29641 | NA | IPR021109 Aspartic peptidase domain superfamily | NA | NA | NA |
| g29830 | NA | IPR021109 Aspartic peptidase domain superfamily | NA | NA | NA |
| g4098 | NA | IPR021109 Aspartic peptidase domain superfamily | NA | NA | NA |
| g8048 | NA | IPR027417 P-loop containing nucleoside triphosphate hydrolase | NA | NA | NA |
| g10774 | NA | IPR032675 Leucine-rich repeat domain superfamily | NA | NA | NA |
| g11897 | NA | IPR032675 Leucine-rich repeat domain superfamily | NA | NA | NA |
| g13299 | NA | IPR032675 Leucine-rich repeat domain superfamily | NA | NA | NA |
| g16298 | NA | IPR032675 Leucine-rich repeat domain superfamily | NA | NA | NA |
| g18164 | NA | IPR032675 Leucine-rich repeat domain superfamily | NA | NA | NA |
| g22141 | NA | IPR032675 Leucine-rich repeat domain superfamily | NA | NA | NA |
| g26598 | NA | IPR032675 Leucine-rich repeat domain superfamily | NA | SignalP(1-22,cutsite=ASS-VN,prob=0.3259) | NA |
| g3297 | NA | IPR032675 Leucine-rich repeat domain superfamily | NA | NA | NA |
| g5732 | NA | IPR032675 Leucine-rich repeat domain superfamily | NA | NA | NA |
| g12039 | NA | IPR032675 Leucine-rich repeat domain superfamily;IPR036047 F-box-like domain superfamily | GO:0005515 - protein binding | NA | NA |
| g23025 | NA | IPR032675 Leucine-rich repeat domain superfamily;IPR036047 F-box-like domain superfamily | GO:0005515 - protein binding | NA | NA |
| g2149 | NA | IPR033341 Spindle and kinetochore-associated protein 3 | GO_component: GO:0000940 - outer kinetochore ;GO_process: GO:0007059 - chromosome segregation | NA | NA |
| g18413 | NA | IPR036047 F-box-like domain superfamily | GO:0005515 - protein binding | NA | Cluster_1 |
| g9175 | NA | IPR036047 F-box-like domain superfamily | GO:0005515 - protein binding | NA | NA |
| g7880 | NA | IPR036612 K Homology domain, type 1 superfamily | GO:0003723 - RNA binding | NA | NA |
| g9132 | NA | IPR036910 High mobility group box domain superfamily | NA | NA | NA |
| g11050 | PF20147 | IPR045379 Crinkler effector protein, N-terminal | NA | SignalP(1-22,cutsite=ANS-AE,prob=0.9158) | NA |
| g27622 | PF20147 | IPR045379 Crinkler effector protein, N-terminal | NA | NA | NA |
| g10200 | NA | NA | NA | NA | NA |
| g10201 | NA | NA | NA | NA | NA |
| g10222 | NA | NA | NA | NA | NA |
| g10241 | NA | NA | NA | NA | NA |
| g10799 | NA | NA | NA | NA | NA |
| g1096 | NA | NA | NA | NA | NA |
| g1103 | NA | NA | NA | NA | NA |
| g11317 | NA | NA | NA | NA | NA |
| g11323 | NA | NA | NA | NA | NA |
| g11840 | NA | NA | NA | NA | NA |
| g11842 | NA | NA | NA | NA | NA |
| g11845 | NA | NA | NA | NA | NA |
| g11997 | NA | NA | NA | NA | NA |
| g12100 | NA | NA | NA | NA | NA |
| g12981 | NA | NA | NA | NA | NA |
| g13402 | NA | NA | NA | NA | NA |
| g13405 | NA | NA | NA | NA | NA |
| g13418 | NA | NA | NA | NA | NA |
| g13517 | NA | NA | NA | NA | NA |
| g13522 | NA | NA | NA | NA | NA |
| g13526 | NA | NA | NA | NA | NA |
| g13594 | NA | NA | NA | NA | NA |
| g13605 | NA | NA | NA | NA | NA |
| g1363 | NA | NA | NA | NA | NA |
| g1407 | NA | NA | NA | NA | NA |
| g14286 | NA | NA | NA | NA | NA |
| g1489 | NA | NA | NA | NA | NA |
| g14923 | NA | NA | NA | NA | NA |
| g14925 | NA | NA | NA | NA | NA |
| g14926 | NA | NA | NA | NA | NA |
| g14937 | NA | NA | NA | NA | NA |
| g14940 | NA | NA | NA | NA | NA |
| g14996 | NA | NA | NA | NA | NA |
| g15325 | NA | NA | NA | NA | NA |
| g1534 | NA | NA | NA | NA | NA |
| g15380 | NA | NA | NA | NA | NA |
| g15507 | NA | NA | NA | NA | NA |
| g16101 | NA | NA | NA | NA | NA |
| g1611 | NA | NA | NA | NA | NA |
| g16292 | NA | NA | NA | NA | NA |
| g16308 | NA | NA | NA | NA | NA |
| g16442 | NA | NA | NA | NA | NA |
| g16519 | NA | NA | NA | NA | NA |
| g16614 | NA | NA | NA | NA | NA |
| g16789 | NA | NA | NA | NA | NA |
| g17036 | NA | NA | NA | NA | NA |
| g1721 | NA | NA | NA | SignalP(1-20,cutsite=VQA-NG,prob=0.8413) | NA |
| g18008 | NA | NA | NA | NA | NA |
| g18009 | NA | NA | NA | NA | NA |
| g18010 | NA | NA | NA | NA | NA |
| g18022 | NA | NA | NA | NA | NA |
| g18368 | NA | NA | NA | NA | NA |
| g18369 | NA | NA | NA | NA | NA |
| g1840 | NA | NA | NA | SignalP(1-21,cutsite=VQA-NG,prob=0.8636) | NA |
| g18971 | NA | NA | NA | NA | NA |
| g18987 | NA | NA | NA | NA | NA |
| g19141 | NA | NA | NA | NA | NA |
| g19412 | NA | NA | NA | NA | NA |
| g19997 | NA | NA | NA | NA | NA |
| g20129 | NA | NA | NA | NA | NA |
| g20183 | NA | NA | NA | SignalP(1-21,cutsite=AST-SY,prob=0.3526) | NA |
| g2073 | NA | NA | NA | NA | NA |
| g20818 | NA | NA | NA | NA | NA |
| g20924 | NA | NA | NA | NA | NA |
| g20927 | NA | NA | NA | NA | NA |
| g21123 | NA | NA | NA | NA | NA |
| g21144 | NA | NA | NA | NA | NA |
| g21284 | NA | NA | NA | NA | NA |
| g21304 | NA | NA | NA | NA | NA |
| g21324 | NA | NA | NA | NA | NA |
| g21388 | NA | NA | NA | NA | NA |
| g21860 | NA | NA | NA | NA | NA |
| g21866 | NA | NA | NA | NA | NA |
| g21867 | NA | NA | NA | NA | NA |
| g21962 | NA | NA | NA | NA | NA |
| g21964 | NA | NA | NA | NA | NA |
| g21965 | NA | NA | NA | NA | NA |
| g22069 | NA | NA | NA | SignalP(1-23,cutsite=AHG-DY,prob=0.5627) | NA |
| g22121 | NA | NA | NA | NA | NA |
| g22246 | NA | NA | NA | NA | NA |
| g22556 | NA | NA | NA | NA | NA |
| g22558 | NA | NA | NA | NA | NA |
| g22640 | NA | NA | NA | NA | NA |
| g22752 | NA | NA | NA | NA | NA |
| g23129 | NA | NA | NA | NA | NA |
| g23281 | NA | NA | NA | SignalP(1-19,cutsite=VNG-IY,prob=0.5919) | NA |
| g23339 | NA | NA | NA | NA | NA |
| g23873 | NA | NA | NA | NA | NA |
| g23956 | NA | NA | NA | NA | NA |
| g23958 | NA | NA | NA | NA | NA |
| g23959 | NA | NA | NA | NA | NA |
| g23970 | NA | NA | NA | NA | NA |
| g2402 | NA | NA | NA | NA | NA |
| g24126 | NA | NA | NA | NA | NA |
| g24136 | NA | NA | NA | NA | NA |
| g24467 | NA | NA | NA | NA | NA |
| g24484 | NA | NA | NA | NA | NA |
| g24551 | NA | NA | NA | NA | NA |
| g24596 | NA | NA | NA | NA | NA |
| g24893 | NA | NA | NA | NA | NA |
| g24975 | NA | NA | NA | NA | NA |
| g25140 | NA | NA | NA | NA | NA |
| g25359 | NA | NA | NA | NA | NA |
| g25740 | NA | NA | NA | NA | NA |
| g25751 | NA | NA | NA | NA | NA |
| g2617 | NA | NA | NA | NA | NA |
| g2618 | NA | NA | NA | NA | NA |
| g2620 | NA | NA | NA | NA | NA |
| g2622 | NA | NA | NA | NA | NA |
| g26308 | NA | NA | NA | NA | NA |
| g26336 | NA | NA | NA | NA | NA |
| g26671 | NA | NA | NA | NA | NA |
| g26678 | NA | NA | NA | SignalP(1-20,cutsite=IHS-FP,prob=0.8053) | NA |
| g26683 | NA | NA | NA | NA | NA |
| g26711 | NA | NA | NA | NA | NA |
| g26713 | NA | NA | NA | NA | NA |
| g26721 | NA | NA | NA | NA | NA |
| g26733 | NA | NA | NA | NA | NA |
| g26739 | NA | NA | NA | NA | NA |
| g26820 | NA | NA | NA | NA | NA |
| g2695 | NA | NA | NA | NA | NA |
| g26968 | NA | NA | NA | NA | NA |
| g2707 | NA | NA | NA | NA | NA |
| g27276 | NA | NA | NA | NA | NA |
| g27384 | NA | NA | NA | NA | NA |
| g27618 | NA | NA | NA | NA | NA |
| g27628 | NA | NA | NA | NA | NA |
| g27760 | NA | NA | NA | NA | NA |
| g2836 | NA | NA | NA | NA | NA |
| g2841 | NA | NA | NA | NA | NA |
| g28473 | NA | NA | NA | NA | NA |
| g2862 | NA | NA | NA | NA | NA |
| g2863 | NA | NA | NA | NA | NA |
| g2865 | NA | NA | NA | NA | NA |
| g2869 | NA | NA | NA | NA | NA |
| g28805 | NA | NA | NA | NA | NA |
| g28817 | NA | NA | NA | NA | NA |
| g29599 | NA | NA | NA | NA | NA |
| g29637 | NA | NA | NA | NA | NA |
| g29651 | NA | NA | NA | NA | NA |
| g29810 | NA | NA | NA | NA | NA |
| g29814 | NA | NA | NA | NA | NA |
| g29832 | NA | NA | NA | NA | NA |
| g29848 | NA | NA | NA | NA | NA |
| g3005 | NA | NA | NA | NA | NA |
| g3013 | NA | NA | NA | NA | NA |
| g30148 | NA | NA | NA | NA | NA |
| g30951 | NA | NA | NA | NA | NA |
| g31061 | NA | NA | NA | NA | NA |
| g31273 | NA | NA | NA | NA | NA |
| g3131 | NA | NA | NA | NA | NA |
| g3373 | NA | NA | NA | NA | NA |
| g3412 | NA | NA | NA | NA | NA |
| g3426 | NA | NA | NA | NA | NA |
| g3630 | NA | NA | NA | NA | NA |
| g3634 | NA | NA | NA | NA | NA |
| g3734 | NA | NA | NA | NA | NA |
| g3821 | NA | NA | NA | NA | NA |
| g4053 | NA | NA | NA | NA | NA |
| g4112 | NA | NA | NA | NA | NA |
| g4114 | NA | NA | NA | NA | NA |
| g4176 | NA | NA | NA | NA | NA |
| g4183 | NA | NA | NA | NA | NA |
| g4251 | NA | NA | NA | NA | NA |
| g4272 | NA | NA | NA | NA | NA |
| g4296 | NA | NA | NA | NA | NA |
| g4310 | NA | NA | NA | NA | NA |
| g4432 | NA | NA | NA | NA | NA |
| g4512 | NA | NA | NA | NA | NA |
| g452 | NA | NA | NA | NA | NA |
| g4798 | NA | NA | NA | NA | NA |
| g5087 | NA | NA | NA | NA | NA |
| g5088 | NA | NA | NA | NA | NA |
| g5089 | NA | NA | NA | NA | NA |
| g5093 | NA | NA | NA | NA | NA |
| g5161 | NA | NA | NA | NA | NA |
| g533 | NA | NA | NA | NA | NA |
| g538 | NA | NA | NA | NA | NA |
| g5536 | NA | NA | NA | NA | NA |
| g6028 | NA | NA | NA | NA | NA |
| g6862 | NA | NA | NA | NA | NA |
| g7479 | NA | NA | NA | NA | NA |
| g7501 | NA | NA | NA | NA | NA |
| g7530 | NA | NA | NA | NA | NA |
| g769 | NA | NA | NA | NA | NA |
| g7740 | NA | NA | NA | NA | NA |
| g7768 | NA | NA | NA | NA | NA |
| g784 | NA | NA | NA | NA | NA |
| g7943 | NA | NA | NA | NA | NA |
| g7994 | NA | NA | NA | NA | NA |
| g8032 | NA | NA | NA | NA | NA |
| g8109 | NA | NA | NA | NA | NA |
| g8138 | NA | NA | NA | NA | NA |
| g8147 | NA | NA | NA | NA | NA |
| g8741 | NA | NA | NA | NA | NA |
| g8788 | NA | NA | NA | NA | NA |
| g8789 | NA | NA | NA | NA | NA |
| g8791 | NA | NA | NA | NA | NA |
| g8827 | NA | NA | NA | NA | NA |
| g8917 | NA | NA | NA | NA | NA |
| g8938 | NA | NA | NA | NA | NA |
| g8945 | NA | NA | NA | NA | NA |
| g8996 | NA | NA | NA | NA | NA |
| g9017 | NA | NA | NA | NA | NA |
| g9142 | NA | NA | NA | NA | NA |
